# Favorable alleles mining for gelatinization temperature, gel consistency and amylose content in *Oryza sativa* by association mapping

**DOI:** 10.1186/s12863-019-0735-y

**Published:** 2019-03-19

**Authors:** Hui Wang, Shangshang Zhu, Xiaojing Dang, Erbao Liu, Xiaoxiao Hu, Moaz Salah Eltahawy, Imdad Ullah Zaid, Delin Hong

**Affiliations:** 1 Nanjing Agricultural University, Nanjing, 210095 China; 2 State Key Laboratory of Crop Genetics and Germplasm Enhancement, Nanjing Agricultural University, Nanjing, 210095 China

**Keywords:** *Oryza sativa*, Linkage disequilibrium,·Genome-wide association mapping, Phenotypic and genetic diversities, Gelatinization temperature, Gel consistency, Amylose content

## Abstract

**Background:**

Improving the gelatinization temperature (GT), gel consistency (GC) and amylose content (AC) for parental grain eating and cooking qualities (ECQs) are key factors for enhancing average grain ECQs for hybrid japonica rice.

**Results:**

In this study, a genome-wide association mapping (GWAS) for ECQs was performed on a selected sample of 462 rice accessions in 5 environments using 262 simple sequence repeat markers. We identified 10 loci and 27 favorable alleles for GT, GC and AC, and some of these loci were overlapped with starch synthesis-related genes. Four SSR loci for the GT trait were distributed on chromosomes 3, 5, 8, and 9, of which two SSR loci were novel. Two SSR loci associated with the GC trait were distributed on chromosomes 3 and 6, although only one SSR locus was novel. Four SSR loci associated with the AC trait were distributed on chromosomes 3, 6, 10, and 11, of which three SSR loci were novel. The novel loci RM6712 and RM6327 were simultaneously identified in more than 2 environments and were potentially reliable QTLs for ECQs, with 15 parental combinations being predicted. These QTLs and parental combinations should be used in molecular breeding to improve japonica rice average ECQs.

**Conclusions:**

Among the 10 SSR loci associated with GT, GC and AC for grain ECQs detected in 27 favorable alleles, the favorable allele RM3600-90bp on chromosome 9 could significantly reduce GT, RM5753-115bp on chromosome 6 could significantly increase GC, and RM6327-230bp on chromosome 11 could significantly reduce AC in hybrid japonica rice mixed rice samples.

**Electronic supplementary material:**

The online version of this article (10.1186/s12863-019-0735-y) contains supplementary material, which is available to authorized users.

## Background

Rice (*Oryza sativa* L.) is one of the most important food crops grown worldwide for more than half of the world’s population [1]. The success of hybrid rice in China was a significant step for improving rice grain yield, with the yield of hybrid rice being 15-20% higher than that of high-yielding conventional varieties [2]. During the last 20 years, the annual planting acreage for hybrid indica rice in China accounted for approximately 80% of the total indica rice area, while hybrid japonica rice only accounted for approximately 5% of the total japonica rice area [3]. One of the main reasons for the slow development of hybrid japonica rice is that the grain eating and cooking qualities (ECQs) are not as good as conventional japonica rice [4]. For hybrid rice cultivars, although F_1_ hybrid rice plant populations are consistent, grains harvested from commercial F_1_ rice hybrid plants represent the F_2_ seed generation and are segregated for some grain characteristics. Thus, we must consider the effect of this segregation on rice ECQs [5].

Rice ECQs mainly include gelatinization temperature (GT), gel consistency (GC) and amylose content (AC) [6]. People in different countries or regions have different requirements for rice ECQs. Consumers north of the Yangtze River in China prefer whole-grain milled rice with lower GT, softer GC and lower AC [7]. GT, indicated by the alkali spreading score (ASS), is a significant characteristic that reflects rice cooking time [8]. Approximately twenty QTLs controlling GT, which are located on chromosomes of 1, 2, 5, 6, 7, 8, 10, 11 and 12, have been reported [9–15]. The gene *ALK*, which is also as known as *SSII-3* (chromosome 6: 6748363-6753338), has been reported to regulate rice GT [9, 11, 16]. Map-based cloning of rice *ALK* shows that the gene encodes soluble starch synthase II (*SSSII*) [11]. GC is a fit indicator to measure cooked rice flour cold paste-viscosity, especially among grains with high amylose contents [17]. Twenty-two QTLs were detected as controlling GC, and they reside on chromosomes 1, 2, 6 and 7 [12, 18, 19]. AC is recognized as the most important factor among rice ECQs, as it affects grain texture, is directly related to water absorption and other characteristics of cooked grains and is wildly used to classify rice ECQs [8]. Fifty-one QTLs controlling AC were detected [12, 14, 15, 20, 21]. *W*_*X*_ located on chromosome 6 encodes granule-bound starch synthase (GBSSI), and two alleles, *Wx*^*a*^ and *Wx*^*b*^, have also been reported at this site [22]. Isshiki et al. (2000, 2008) reported the genetic regulation of the amylose synthesis pathway in dull mutants [23, 24]. Additionally, *Du1* (chromosome 7), *Du2* and *Du3* (chromosome 2), *Du4* and *Du5* (chromosome 4), and *Du12-t* on the long arm of chromosome 6 were also reported as controlling AC [20, 23, 25–28]. The regions surrounding the *Wx* locus are highly diverse, which indicates complex histories for the selection for different types of rice ECQs [29, 30].

The ECQs have been widely studied in cereals. Mo (1988) first showed that the inheritance of rice ECQs is complicated by the maternal plants or cytoplasm effects, and is mainly controlled by triploid endosperm genotypes [31]. They were determined througha mating design and its corresponding statistical method, through which a model was used to independently test the genetic effects of the endosperm and maternal genotype, as well as effects of cytoplasmic differences in the segregated generations [32]. Xu et al. (1995) reported that the inheritance of rice ECQs was mainly controlled by the triploid endosperm genotype with slight cytoplasmic effects [33]. Although the aforementioned models were used to identify the genetic expression of quality traits in cereal, these reports usually used heterozygous individuals from the segregated progeny. He et al. (1999) established a doubled haploid (DH) population to identify nine QTLs for rice ECQs; he proposed that a permanent segregation population can provide sufficiently homozygous generations for the analysis of rice ECQs [10]. Aluko et al. (2004) and Bao et al. (2002) suggested that minor QTLs for rice ECQs can be detected for the near-isogenic line (NIL) population [14, 34]. Though some QTLs for rice ECQs have been successful analyzed in the abovementioned studies, traditional biparental segregated populations showed several limitations, including finite genetic variation and recombination [35]. Association analysis is a method based on the linkage disequilibrium to detect the correlations between phenotypic variation and genotype [36]. Additionally, compared with traditional QTL mapping, association analysis that occurs in the natural population has the advantage of more recombination probabilities and carries on the validly complement for segregation population; for example, previous studies showed that linkage disequilibrium mapping improves the QTL mapping resolution by 500 times [37]. With the rapid development of plant genomics, association analysis has enabled researchers to develop polymorphisms and locate alleles in the genome [38]. Therefore, it has become a valuable and powerful method to exploit SSR markers for complex quantitative traits [39–42]. Borba et al. (2010) and Xu et al. (2016) presented to use the marker-trait associations for mining of alleles associated with rice grain quality traits [15, 43]. Although a few reports have focused on association mapping for rice ECQs, the geographical and ecological distribution of the experimental populations was insufficient and without abundant genetic diversity, and the extent of linkage disequilibrium (LD) was low. This technique has allowed researchers to exploit natural diversity and mine valuable genes in the genome.

A natural population of 462 accessions derived from seven different ecological regions was developed in our laboratory for QTL identification [44–46]. This population has several advantages over the permanent segregation population for detecting QTLs and mining valuable genes in the genome. On one hand, the natural population contains sufficient germplasm resources that are suitable for mining more favorable alleles for rice grain quality traits. In contrast, the homozygotic genotype of each accession in the natural population could simplify the QTL detection of grain quality traits and inconsiderate of the effects of triploid endosperm genotypes, both maternal and cytoplasmic.

In this study, an experimental natural population containing a set of 462 rice accessions and 262 SSR marker pairs were used to conduct association mapping for rice ECQs on a genome-wide scale through five environments. The purposes were to detect QTLs significantly associated with the ECQs, mine favorable alleles, and then explore the parental combinations for improving rice ECQs.

## Methods

### Plant materials and field planting

The 462 rice varieties originating from different regions of Asia were used as the plant materials in this study represent a subset of our previous report [44]. Among the varieties, 341 were from China and 121 were from Vietnam (Additional file 1: Table S1). In the summers of 2011, 2012 and 2013, the 462 accessions were grown in the five environments, E1 to E5, which included three locations, Nanjing, Yuanyang and Xinyang (Additional file 2: Table S2). Each entry plot was composed of eight rows with eight plants per row. All plots were arranged with a randomized complete block design and two replicates per environment.

### Evaluations of GT (ASS), GC and AC

After harvest, the rough rice grains were air-dried under natural sunshine conditions, stored at room temperature for 3 months, and then kept in a refrigerator at 4°C for reserve. Then, the rough rice was milled into brown rice using a rice huller (JLGJ45, manufactured by the Taizhou Food and Oil Machinery Factory, Zhejiang, China). The brown rice was then processed into milled rice using a rice milling machine (JNMJ3, manufactured by the Huangyan Food and Oil Machinery Factory, Zhejiang, China) as follows. Twenty grams of brown rice per accession was placed into the miller machine and milled for 70 s. One half of the polished head rice was used for measuring the GT and the other half was ground into a fine powder using the flour mill (FW100, Tianjin Tai Si Te Instrument Inc, China) and kept in each Ziploc bags for measuring the GC and AC.

The GT was estimated indirectly as the alkali spreading score (ASS) using the method from Little et al. (1958) [47] with minor modifications. Briefly, 6 grains of intact milled white rice from each accession were placed in a 60-mm petri dish with 10 ml of 1.7% KOH. The samples were separated from each other using forceps and incubated at 30±0.5°C for 23 h to allow the grains to spread. The spreading score of the grains was recorded by visual assessment as described by Jennings et al. [48]. Based on the appearance of the endosperm and the degree of dispersion, an ASS score from 1-7 was recorded. Unaffected or slightly swollen endosperms were recorded 1, while those that completely disappeared were recorded as 7 (Fig. 2b). GT values are inversely related to the ASS score. The following are the three classes of GT: ASS from 1 to 3 grade is high GT (> 75°C), from 4 to 5 grade is intermediate GT (70-74°C), and from 6-7 grade is low GT (<70°C) [6].

The GC was determined according to the method of Cagampang et al. [49]. Briefly, 100 mg rice flour with approximately 12% moisture content was weighed in a test tube, to which 0.2 ml of 95% ethanol containing thymol blue was added and gently shaken to prevent clumping of the powder during gelatinization. Two milliliters of 0.2 mol/L KOH were added and vortexed thoroughly. The tubes were covered with glass marbles and boiled in a water bath to reflux for 8 min. After cooling down at room temperature for 5~10 min, the tubes were placed on ice for 20 min and then laid down horizontally on a table surface for 1 h. The gel length (mm), which was measured as the distance from the bottom of the tube to the front of the gel migration, is a measurement of GC. A longer gel correlates with a higher GC value.

The AC of the rice grains was measured using an automatic microplate spectrophotometer (TECAN Infinite 200 Pro, Untersbergstrasse 1A, Austria, Additional file 3: Figure S1A) according to the method described of Zhu et al. [50]. Briefly, 50 mg of test sample flour was weighed and added to a test tube, followed by the addition of 0.5 ml 95% ethanol and 1.5 ml of 3 mol/L NaOH. The tube was placed at 30°C overnight (12-16 h) after being fully skimmed and shaken lightly with distilled water to increase the volume to 40 ml. 10 μl of the mixture was added to a 96-well ELISA plate; the remaining reaction solution consisted of 2 μl of 1 mol/L acetic acid, 3 μl of 2% I_2_-KI, and 185 μl distilled water to bring the final volume to 200 μl (Additional file 3: Figure S1B). We then used a microplate spectrophotometer to automatically record the optical density value (OD) at 620 nm, which was shown on the computer monitor (Additional file 3: Figure S1C). The AC was calculated using a standard curve.

### Phenotypic data analysis

The phenotypic data statistical analyses were performed using the SPSS software (IBM Institute Inc., Armonk, NY, USA). The broad sense heritability (${H}_B^2$) was calculated by using the formula ${H}_B^2={\upsigma}_g^2/\left({\upsigma}_g^2+{\upsigma}_e^2/n\right)$ [51], where ${\upsigma}_g^2$ represents the genetic variance, ${\upsigma}_{\mathrm{e}}^2$ represents the error variance, and *n* represents the number of replications.

### SSR marker genotyping

Young leaves at the tillering stage were collected from each plant of each accession and were genotyped using SSR molecular markers. Genomic DNA was extracted following the methods described by Tai and Tanksley [52]. A total of 262 SSR markers selected from 12 rice chromosomes were used for genotyping the 462 accessions studied. PCR process, PCR product separation and record of band size followed the methods described by [44].

### Genotypic data analysis

The population structure of 462 accessions was analyzed with three methods. Firstly, the Bayesian clustering method, using the STRUCTURE 2.3 software to implement [53, 54]. Secondly, the neighbour-joining method, using the PowerMarker (version 3.25) software to carry out the polymorphism information content (PIC), then the neighbor-joining tree was built based on Nei's genetic distance and framed using the MEGA 5.0 software [55]. The calculations followed the same method as those described in [44]. Finally, the principal component analysis (PCA) for each subpopulation was calculated by the RTM-GWAS program with default parameters by using the 262 SSR markers [56]. The coefficient of genetic differentiation (*F*_ST_) was estimated to measure the fixation of different alleles in different subpopulations based on the method that was proposed by Weir and Hill [57], and the computing process was completed using the Arlequin 3.11 software [58].

### Linkage disequilibrium analysis

Prior to the association analysis, data with a minimum allele frequency (MAF) < 5% by filter alignment were removed from the dataset. According to the level of LD and the genetic distance between the markers with intra-chromosomal combinations, the regression equation of LD with genetic distance was calculated by regression analysis. The relationship between LD and genetic distance was observed by plotting LD decay.

### Association analysis

To control for false positives, the *K* (the kinship matrix) + *Q* (population structure matrix) model was used for the genome-wide association mapping based on the mixed linear model (MLM) in the TASSEL 5.0 software [59]. The *Q* matrix was obtained from the STRUCTURE 2.3 analysis results. The *K* matrix was generated from the relatedness analysis results using the kinship matrix function in TASSEL 5.0. The false discovery rate (FDR) was used to control for the proportion of falsely refused hypotheses for significant associations using the Muralidharan correction method [60]. A -LogP value greater than 3 was set as a threshold for strong associations, and the quantile-quantile (Q-Q) and Manhattan plots for significantly associated markers were performed using TASSEL 5.0. Based on the association locus identified, the phenotypic effects of the alleles were estimated by the ‘null allele’ (non-amplified allele) for each locus [61]. The formula used for calculating the phenotypic effect of average positive (negative) allelic effects (AAE^+^ or AAE^-^) followed the same method as described in [44].

## Results

### Phenotypic evaluations

Table 1 shows the mean value, coefficient of variation and heritability in the broad sense (${H}_B^2$) for GT, GC and AC measured in the 462 varieties across the five environments. The CVs ranged from 30.79% for AC to 56.23% for GC, suggesting significant variations in the 3 traits among the accessions across the five environments. Continuous distributions were observed for GT, GC, and AC, and the phenotypic data for all the three traits followed a normal distribution based on the skewness and kurtosis values. A two-way analysis of variance (ANOVA) showed that differences among the 462 accessions for GT, GC and AC were highly significant (*P* < 0.01), indicating that there were a large amount of genetic variation in the natural population. The average ${H}_B^2$ across 5 environments for GT, GC, and AC was 95%, 94% and 98%, respectively.

**Table 1:** Summary statistics of phenotypic performance of 462 rice accessions for GT, GC and AC in five environments

Traits^a^	Environments	Mean±SD ^b^	Maximum	Minimum	CV (%)^c^	Kurtosis	Skewness	*H*^2^_B_ (%)
GT (ASS, grade)	E1	4.65±1.83	7	1	39.35	-0.87	-0.59	95.0
	E2	4.45±1.62	7	1	36.4	-0.48	-0.59	92.7
	E3	4.61±1.77	7	1	38.39	-0.39	-0.84	95.5
	E4	3.95±1.76	7	1	44.56	-1.12	-0.58	94.9
	E5	3.60±1.71	7	1	47.5	-1.29	-0.14	94.6
GC (mm)	E1	53.07±25.4	139	8	47.86	0.3	0.76	94.5
	E2	50.04±25.19	134	14	50.34	0.91	1.02	96.2
	E3	46.09±25.22	134	15	54.72	1.12	1.1	96.0
	E4	44.05±24.77	138	13	56.23	1.87	1.4	92.9
	E5	49.5±25.06	137	15	50.63	1.02	1.05	90.7
AC (%)	E1	18.22±5.93	27.44	0.04	32.55	2.44	-1.55	96.3
	E2	19.37±6.5	30.67	0.81	33.56	1.68	-1.29	92.8
	E3	17.64±5.61	28.62	0.65	31.8	2.61	-1.58	99.7
	E4	18.69±6.64	31.77	0.31	35.53	0.89	-1.12	99.6
	E5	19.36±5.96	29.69	0.28	30.79	3.31	-1.79	99.8

^a^GT (gelatinization temperature) values based on ASS (alkali spreading score) from 1-7; *GC* gel consistency; *AC* amylose content; See Materials and methods for details of parameter calculations

^b^*SD* standard deviation

^c^*CV* Coefficient of variation

**Fig. 1:**
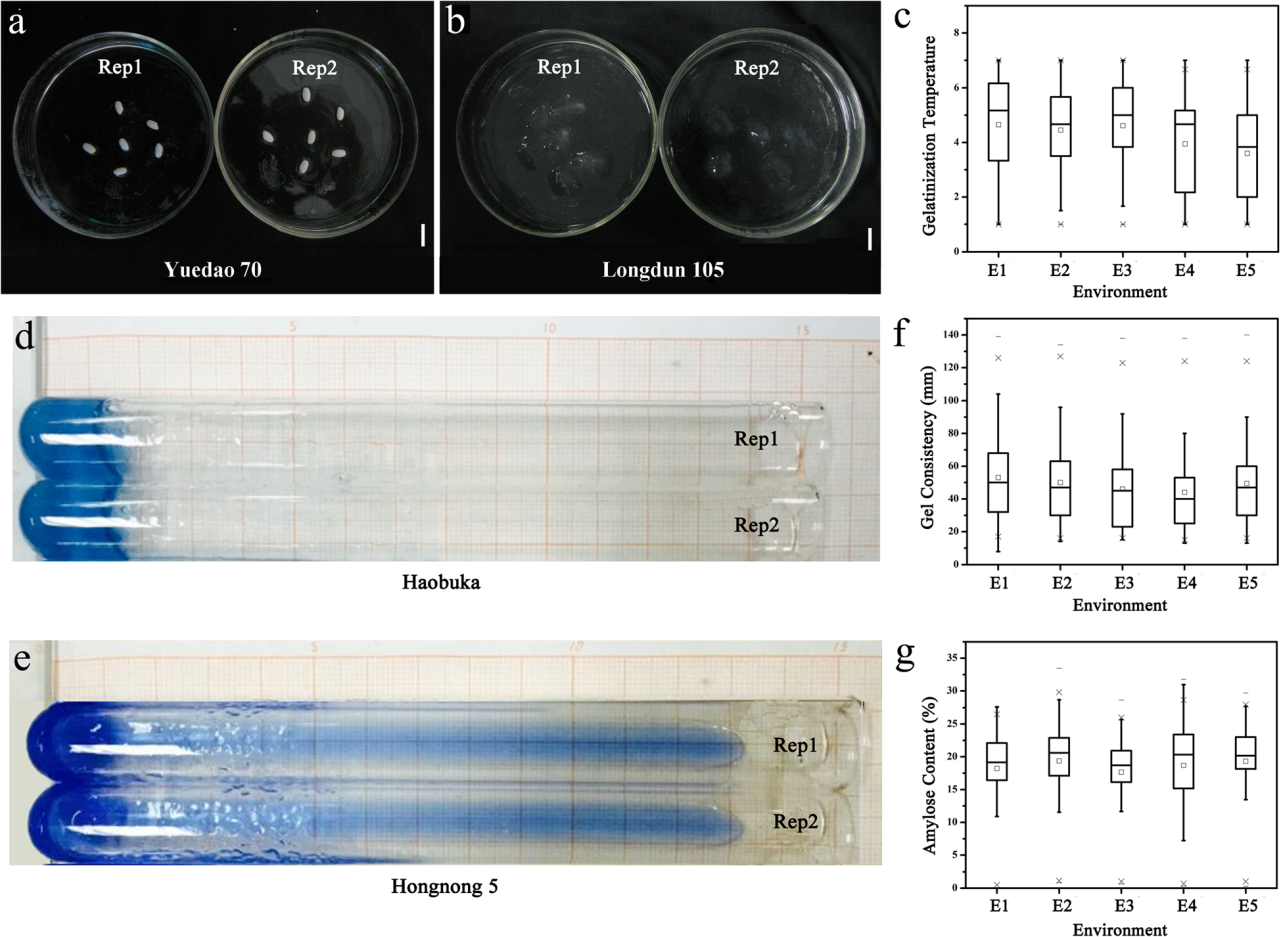
Graphical representation of different typical variety of rice showing the highest and the lowest values in gelatinization temperature which measured by the alkali spreading score (ASS) and gel consistency trait. **a** Rice of the lowest ASS named ‘Yuedao 70’. **b** The highest ASS named ‘Longdun 105’. **c** Summary statistics of gelatinization temperature of 462 rice accessions in five environments. GT values based on the ASS (1-7 grade). **d** The shortest length of gel consistent named ‘Haobuka’. **e** The longest length of gel consistent named ‘Hongnong 5’. **f**-**g** Summary statistics of gel consistency and amylose content of 462 rice accessions in five environments. Box plots span the 95th–fifth percentiles. Bar = 10mm

For the GT trait, the mean ASS values for the 462 cultivars were 4.65, 4.45, 4.61, 3.95 and 3.6, with 95.0, 92.7, 95.5, 94.9 and 94.6% ${H}_B^2$ in the five environments, respectively (Table 1). The graphical representation of the GT for typical accessions is shown in Fig. 1. The minimum alkali spreading score is 1, and its typical accession is ‘Yuedao70’. Its rice grains displayed no cracks or swelling; therefore, it belongs to a type with a high gelatinization temperature (Fig. 1a). In contrast, the maximum ASS value is 7, with ‘Longdun105’ as a typical accession (Fig. 1b). These rice grains are completely dispersed and mixed, demonstrating that it has a lower gelatinization temperature. The performance of the GT trait was mostly stable across the 5 environments, except for the median ASS of (3.6±1.71) in E5, which represents a Xinyang Farm in Henan (32°N, 114°E), indicating GT trait was mainly controlled by genetic factors (Fig. 1c).

The mean GC for the 462 accessions were 53.07, 50.04, 46.09, 44.05 and 49.5mm, with 94.5, 96.2, 96.0, 92.9 and 90.7% ${H}_B^2$, respectively. The shortest gel consistency length was 13 mm, with a typical accession as ‘Haobuka’ that has a hard gel consistency (Fig. 1d). The longest gel consistency length was 134 mm; ‘Hongnong5’ was this carrier accession and it displayed a soft gel consistency (Fig. 1e). The median value of the 462 rice accessions was largely consistent across the different environments, though the GC trait showed slightly lower phenotypic values (44.05±24.77) in E4, which represents a Yuanyang Farm in Henan (35°N, 113°E) (Fig. 1f).

The mean AC for the 462 accessions were 18.22, 19.37, 17.64, 18.69 and 19.36%, with 96.3, 92.8, 99.7, 99.6 and 99.8% ${H}_B^2$, respectively. Figure 1g shows that the performance for the AC trait was stable across the 5 environments and was mainly controlled by genetic factors.

Analysis of variance for the GT, GC and AC traits in the population containing the 462 accessions from 2011-2013 at the Nanjing site indicated extremely significant effects due to the genotype, year, year × genotype, and replications within year for the GC and AC traits, but nonsignificant effects due to the replications within year on GT (0.83) (Additional file 4: Table S3). Additionally, the analysis across three sites for 2013 indicated highly significant effects due to the site, genotype, and site × genotype for the GT and GC traits, but nonsignificant effects due to the replications within site for GT (1.12) and GC (2.01) (Additional file 5: Table S4).

### Population genetic structure analysis

The genetic diversity of the 462 accessions was detected using 262 SSR markers, and the analysis resulted in a total of 2462 alleles being detected. The number of alleles per locus ranged from 2 (at locus RM206 on chromosome11) to 25 (RM7545 on chromosome10), with an average of 9.40 alleles per locus (Additional file 6: Table S5). The average genetic diversity was 0.4685 and ranged from 0.0068 (RM7403 on chromosome 3) to 0.7569 (RM128 on chromosome 1) (Additional file 6: Table S5). The average PIC value was 0.4208 and ranged from 0.0067 (RM7403 on chromosome 3) to 0.7444 (RM128 on chromosome 1) (Additional file 6: Table S5). Furthermore, there were 379 unique alleles (allele frequency < 0.5%), 848 rare alleles (0.5% ≤ allele frequency <5%), 1231 polymorphic alleles (5% ≤ allele frequency ≤ 95%) and 4 fixed alleles (allele frequency ≥ 95%) among the 2462 alleles, with corresponding proportions of 15.39%, 34.44%, 50.00% and 0.16%, respectively.

**Fig. 2:**
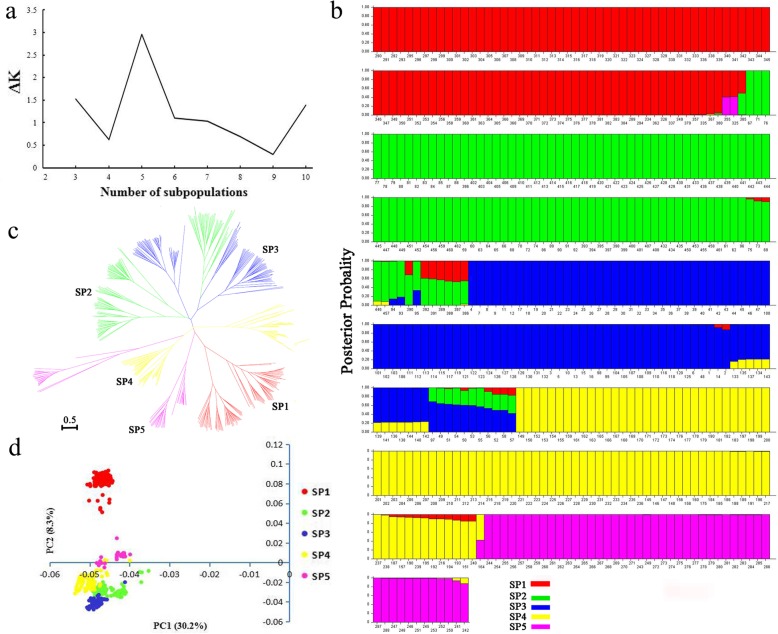
Population structure analysis in 262 rice accessions. **a** Changes in the △k value. **b**) Posterior probability of 262 accessions belonging to five subpopulations calculated by STRUCRURE software. The colored subsections within each vertical bar indicate membership coefficient (Q) of the accession to different clusters. Identified subpopulations are SP1 (red color), SP2 (green color), SP3 (navy blue color), SP4 (yellow color), SP5 (purple color). **c** Neighbor-joining tree for the 262 accessions based on Nei’s genetic distance. **d** Principal components analysis (PCA) for 462 accessions and referance accessions genotyped with 262 SSR markers

An analysis of model-based population structure provided evidence for a significant population structure among the 462 rice accessions. The log-likelihood values increased with the increase in the model parameter *K*; thus, the *ΔK* as the diagnostic criterion was used to determine a suitable value for *K*. The highest *ΔK* value was obtained at *K* = 5 (Fig. 2a). The 462 accessions could be divided into five subpopulations from SP1 to SP5 (Fig. 2b). The information of each accession belonging to each subpopulation (*Q* matrix) is summarized in Additional file 1: Table S1. The neighbor-joining tree constructed based on the Nei's genetic distances showed that the entire population was clearly divided into 5 subpopulations (Fig. 2c), being basically consistent with the results from the Structure analysis. Furthermore, principal component analysis (PCA) plots clearly differentiated the whole population into five subpopulations (Fig. 2d), which corresponded with the population structure results.

**Fig. 3:**
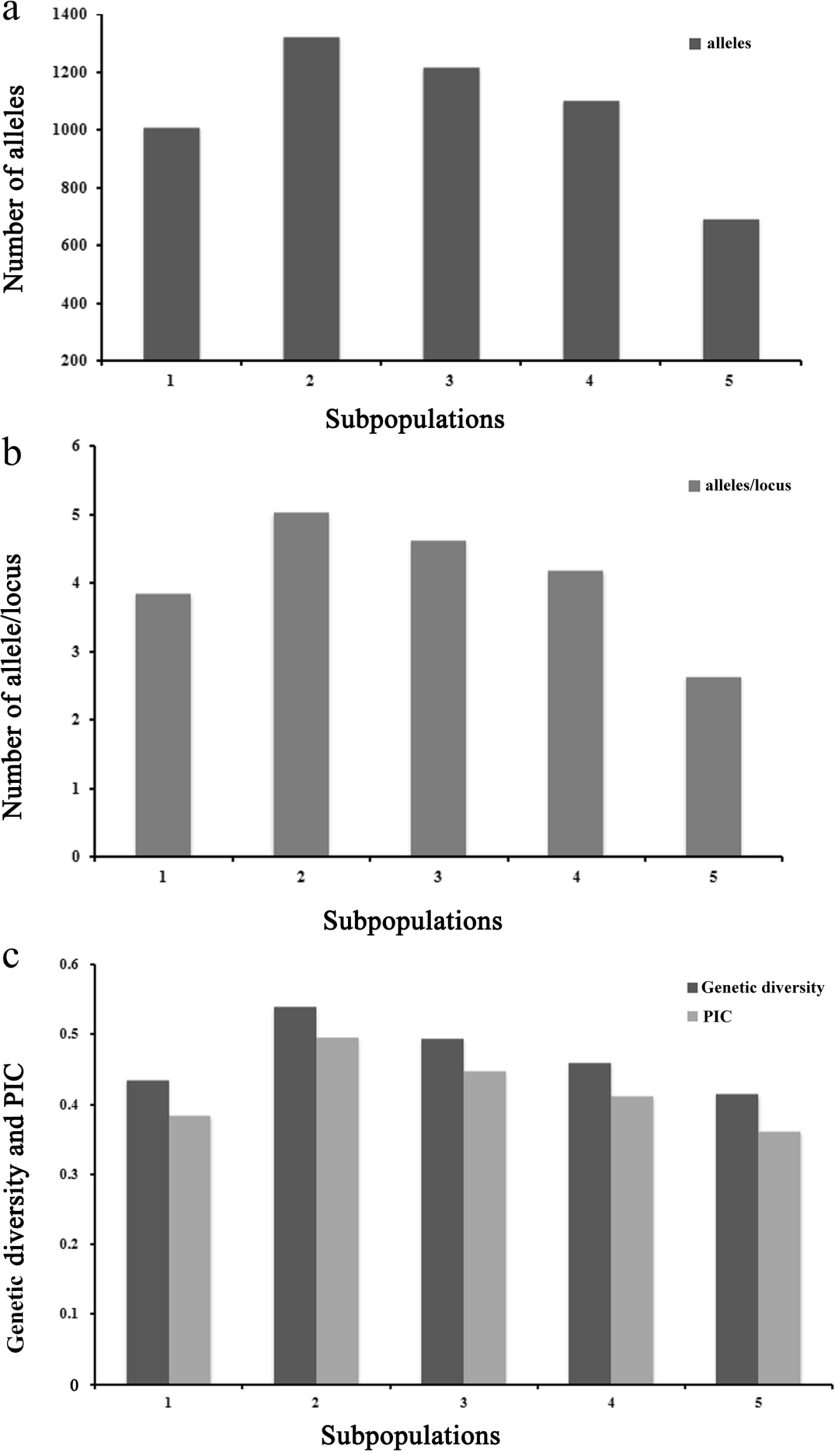
Genetic diversity analysis for the five subpopulations. **a** Number of alleles. **b** Number of alleles/locus in the five subpopulations. **c** Genetic diversity and PIC value in the five subpopulations

Among the five subpopulations, the average pairwise *F*_ST_ value was 0.3827 (Table 2). The pairwise value of *F*_ST_ ranged from 0.2766 (between SP2 and SP3) to 0.4356 (between SP1 and SP5), and the corresponding Nei’s genetic distance between SP2 and SP3 was the lowest (0.3906). SP2 had the highest gene diversity of 0.5394, with a total of 1323 alleles or 5.03 alleles per locus and a PIC value of 0.4954, followed by SP3 with a genetic diversity of 0.4938 and 1215 alleles or 4.62 alleles per locus and a PIC value of 0.4487 (Fig. 3). In contrast, SP5 had the lowest gene diversity of 0.4149, with 2.63 alleles per locus and a PIC value of 0.3621 (Fig. 3). Molecular variance analysis (AMOVA) showed that 34.48% of the total genetic variation occurred between the subpopulations, while 65.52% occurred in the subpopulations (Additional file 7: Table S6). These results suggest a high degree of genetic differentiation among the five subpopulations.

**Table 2:** Pairwise *F*ST and Nei’s genetic distance among the five subpopulations

Subpopulation	1	2	3	4	5
1		0.5307	0.5605	0.5200	0.5414
2	0.3796		0.3906	0.4876	0.6049
3	0.4130	0.2766		0.4254	0.6268
4	0.4305	0.3456	0.3100		0.5328
5	0.4356	0.3901	0.4236	0.4228	

Nei’s genetic distance is above the diagonal, and the pairwise *F*_ST_ is below the diagonal. All of the *F*_ST_ values are significant (*p*<0.05)

### Linkage disequilibrium analysis

A linkage disequilibrium analysis was performed for the natural population varieties. Among the 34,191 pairs, 5,305 pairs showed LD (based on *D′*, P< 0.05), including the combinations of inter- and intra-chromosomal. Among the 5 subpopulations, the lowest percentage of average *D′* was found in SP2 (0.673) (Table 3), indicating that accessions of SP2 might be subjected to intensive artificial selection. The highest *D′* was found in SP5 (0.787) (Table 3). Regression linear analysis between the *D′* value and genetic distance of the syntenic loci showed that the 5 subpopulations followed the formula *y* = *b* ln *x* + *c*. The minimum LD decay distances for SP1-SP5 were 41.8 cM, 79.3 cM, 83.3 cM, 76.4 cM and 63.4cM, respectively. We observed that SP1 had the fastest decay, while SP3 demonstrated the slowest decay velocity among the five subpopulations (Additional file 8: Figure S2).

**Table 3:** Comparison of D′ values for pair-wise SSR loci in each subpopulation

Cluster	No. of significant LD^a^	Ratio^b^ (%)	Frequency of *D*′^c^ value (*P* < 0.05)					Means
	locus pairs		0–0.2	0.2–0.4	0.4-0.6	0.6-0.8	0.8-1.0	of *D*′
POP1	927	2.7	14	154	195	173	391	0.692
POP2	1330	3.9	3	159	316	406	446	0.673
POP3	1137	3.3	0	31	169	349	588	0.786
POP4	1078	3.2	1	50	181	336	510	0.751
POP5	833	2.4	0	24	153	207	449	0.787
	5305							

^a^LD means linkage disequilibrium

^b^Ratio between the number of significant LD locus pairs and total number of LD locus pairs

^c^D′ means standardized disequilibrium coefficients

### Identification of the SSR marker loci associated with GT and favorable alleles

**Fig. 4:**
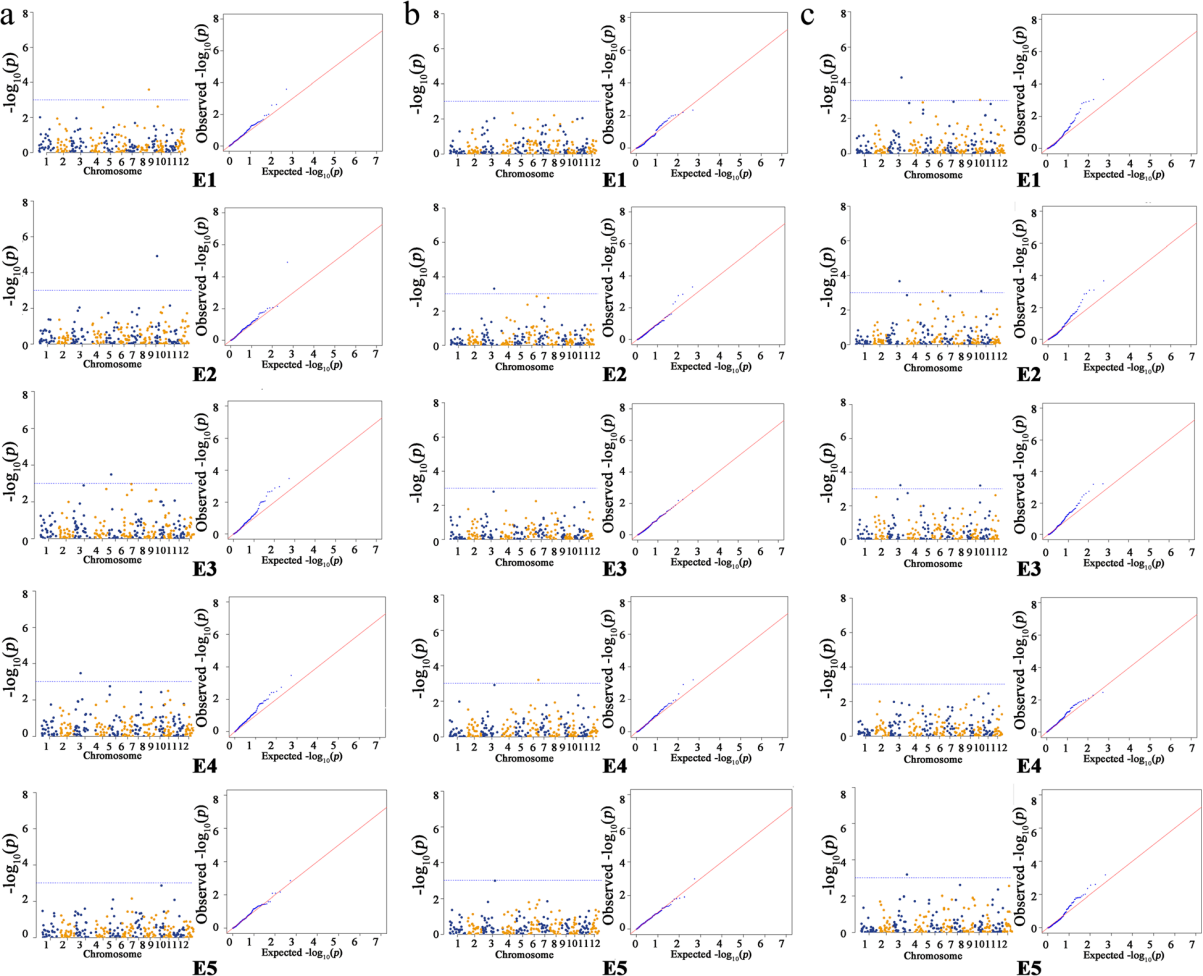
Manhattan and quantile-quantile plots of GWAS studies for GT, GC and AC with mixed linear model (MLM) in the five environments. **a** GT. **b** GC. **c** AC

Four loci (RM232, RM267, RM264, and RM3600) for GT (FDR< 0.05, -LogP≥ 3) were detected on chromosomes 3, 5, 8, and 9, respectively, and had a PVE range from 7.04% to 10.12%. The -LogP values of these loci were 3.48, 3.48, 3.60 and 4.92 in E4, E3, E1 and E2, respectively (Table 4, Fig. 4a). Among them, RM3600 (Chr.9: 17107752-17107843) detected in E2 had corresponding maximum -LogP, PVE and AAE^+^ values of 4.92, 10.12% and 1.38, respectively (Table 4, Fig. 4a).

**Table 4:** Marker–trait associations with –LogP value ≥ 3.0, their equivalent false discovery rate (FDR), proportion of phenotypic variance explained (PVE), and the average allele effects (AAE) across five environments for GT (ASS), GC and AC traits

Traits	No.	SSR marker	Chr.	position (bp)^a^	Environment	-LogP	FDR	PVE(%)	AAE^b^
GT	1	RM232	3	8409404-8410886	E4	3.48	0.0022	9.74	0.80
	2	RM267	5	21881317-21881455	E3	3.48	0.0023	7.04	0.66
	3	RM264	8	27926632-27926652	E1	3.60	0.0024	7.43	1.16
	4	RM3600	9	17107752-17107843	E2	4.92	0.0029	10.12	1.38
GC	1	**RM6712**	3	35020004-35020027	E2	3.30	0.0042	7.46	16.60
	2	**RM5753**	6	30452023-30452067	E4	3.20	0.0028	10.54	20.52
AC	1	**RM6712**	3	35020004-35020027	E1	4.27	0.0022	8.83	-3.57
					E2	3.66	0.0022	7.89	-4.56
					E3	3.22	0.0019	7.17	-4.87
					E5	3.17	0.0020	7.17	-4.76
	2	**RM5753**	6	30452023-30452067	E2	3.08	0.0065	10.48	-3.66
	3	RM258	10	17570591-17570612	E1	3.04	0.0043	5.69	-5.28
	4	RM6327	11	364257-364310	E2	3.09	0.0043	12.87	-2.66
					E3	3.19	0.0038	11.39	-2.75

Bold markers represent that they were associated with two traits

^a^The estimated physical position (bp) was inferred from the Gremene (http://www.gramene.org/markers) and NCBI (https://www.ncbi.nlm.nih.gov/Blast.cgi)

^b^Average positive allele effects of SSR loci associated significantly with GT and GC traits, average negative allele effects of SSR loci associated significantly with AC trait

For GT, the alleles with positive phenotypical effect values are considered favorable alleles. Table 5 shows a summary of the top three favorable alleles and their typical carriers. Among the 12 favorable alleles for GT listed in Table 5, the RM3600-90bp allele showed the largest positive phenotypic effect value (2.0 grade) (Table 5). In total, 86 of 462 accessions carried the allele RM3600-90bp, with a typical carrier accession being ‘Longdun105’ (Fig. 1b), which originated in Northeast China.

**Table 5:** Top three favorable marker alleles on each locus detected GT, GC and AC across five environments and typical varieties carrying the allele

Traits	No.	Locus-allele	Chr.	MAF	Environment	PEV	Typical carrier variety
GT (grade)	1	RM232-155	3	0.071	E4	0.63	Feilaifeng
	2	RM232-145	3	0.192	E4	0.67	Wanyedao
	3	RM232-150	3	0.279	E4	0.03	Laowusi
	4	RM267-95	5	0.086	E3	1.11	Xiangnuodao
	5	RM267-125	5	0.106	E3	0.61	Feilaifeng
	6	RM267-120	5	0.104	E3	0.39	Kuobanzhong
	7	RM3600-90	9	0.186	E2	2.00	Longdun105
	8	RM3600-85	9	0.368	E2	1.02	Ligengqing
	9	RM3600-80	9	0.149	E2	0.29	Songjing12
	10	RM264-195	8	0.128	E1	0.97	Shiluqing
	11	RM264-160	8	0.132	E1	0.85	Kunnong8
	12	RM264-130	8	0.091	E1	0.74	Yebaidao
GC (mm)	1	RM6712-95	3	0.190	E2	13.12	Dongnongjingnuo418
	2	RM6712-115	3	0.223	E2	11.12	Baikenuo
	3	RM5753-195	6	0.108	E4	24.02	Shenlenuo
	4	RM5753-115	6	0.071	E4	28.57	Hongnong5
	5	RM5753-130	6	0.069	E4	3.06	Haonuopie
AC (%)	1	RM6712-115	3	0.223	E1	-4.32	Molingjing
					E2	-1.97	Xudao3
					E3	-0.45	Wujing15
					E5	-2.04	Baoxintaihuqing
	2	RM6712-95	3	0.190	E1	-0.68	Dongnongjingnuo418
					E2	-3.11	Munian4
					E3	-1.67	Hangzhounuo
					E5	-1.24	Munian4
	3	RM5753-195	6	0.108	E2	-3.21	Guozinuo
	4	RM5753-115	6	0.071	E2	-2.21	Suyunuo
	5	RM5753-205	6	0.970	E2	-1.13	Suzhouqing
	6	RM258-125	10	0.392	E1	-1.74	Yue109
	7	RM258-140	10	0.247	E1	-0.76	Wandao68
	8	RM258-135	10	0.307	E1	-0.70	Shenlenuo
	9	RM6327-230	11	0.104	E2	-3.78	Jinggunuo
					E3	-0.85	Hangzhounuo
	10	RM6327-175	11	0.149	E2	-2.53	Baimangnuo
					E3	-1.31	Baimangnuo

Note: Fo r GT and GC trait, alleles with positive phenotypic effect value (PEV) are defined favorable alleles, whereas alleles with negative phenotypic effect value are considered as favorable alleles for AC trait

RM232 (Chr.3: 8409404-8410886), which was associated with GT, had the second highest PVE of 9.74% in E4 and -LogP value and AAE^+^ values of 3.48 and 0.8, respectively (Table 4, Fig. 4a). Four alleles (RM232-160bp, -155bp, -150bp, and -145bp) were detected over the 462 accessions (MAF>5%). All four of these alleles were associated with reducing the GT trait, of which the favorable allele RM232-145bp showed a maximum positive PEV (0.67 grade) for reducing the GT, with the typical carrier accession being ‘Wanyedao’ (Table 5).

RM264 (Chr8: 27926632-27926652), which was associated with GT, had a PVE of 7.43% in E1; its -LogP value and AAE^+^ values were 3.6 and 1.16, respectively (Table 4, Fig. 4a). A total number of 7 alleles were detected over the 462 accessions (MAF>5%). RM264-195bp, -160bp, and -130p were associated with reducing the GT trait, while RM264-185bp, -180bp, -165bp, and -140bp were associated with increasing the GT trait. The favorable allele RM264-195bp showed a maximum positive PEV (0.97 grade) for reducing GT, with the typical carrier accession being ‘Shiluqing’ (Table 5).

RM267 (Chr5: 21881317-21881455), which was associated with GT in E3, had a PVE of 7.04% and -LogP and AAE^+^ values of 3.48 and 0.66, respectively (Table 4, Fig. 4a). Seven alleles were detected over the 462 accessions (MAF>5%). RM267-160bp, -125bp, -120bp, and -95bp were associated with reducing the GT trait, while RM267-200bp, -145bp, and -140bp were associated with increasing the GT trait. The favorable allele RM267-95bp showed the maximum positive PEV (1.11 grade) for reducing GT, with the typical carrier accession being ‘Xiangnuodao’ (Table 5).

### Identification of SSR marker loci associated with GC and favorable alleles

Two loci, RM6712 (Chr.3: 35020004-35020027) and RM5753 (Chr.6: 30452023-30452067), were detected as associated with GC (FDR< 0.05, -LogP≥ 3) in E2 and E4, respectively. Among them, RM5753 had a maximum PVE and positive AAE value, which were 10.54% and 20.52, with a -LogP value of 3.2 (Table 4, Fig. 4b).

The alleles with positive effects are considered favorable alleles for the GC trait. Five favorable alleles were detected over the entire population, with 2 in E2 and 3 in E4. The six alleles from RM5753 were detected over the 462 accessions (MAF>5%). RM5753-205bp, -195bp, -130bp, and -115bp were associated with increasing the GC trait, while RM5753-200bp and -135bp were associated with reducing the GC trait. The favorable allele RM5753-115bp showed the maximum positive phenotypic effect value (25.73 mm) for increasing GC (Table 5). In total, 33 of 462 accessions carried the allele RM5753-115bp, with its typical cultivar being ‘Hongnong5’ (Fig. 1e).

RM6712 had the largest -LogP value at 3.3 and a PVE and positive AAE of 7.46% and 16.6, respectively, in E2 (Table 4, Fig. 4b). Four alleles from RM6712 were detected over the 462 accessions (MAF>5%). RM6712-115bp and -95bp were associated with increasing the GC trait, while RM6712-85bp and -80bp were associated with reducing the GC trait. The favorable allele RM6712-95bp displayed the largest effect (13.12 mm) for GC, with ‘Dongnongjingnuo418’ being a typical carrier accession (Table 5).

### Identification of SSR marker loci associated with AC and favorable alleles

Four loci (RM6712, RM5753, RM258 and RM6327) were associated with AC (FDR< 0.05, -LogP≥ 3) and distributed on chromosomes 3, 6, 10 and 11, respectively, with corresponding -Log P values ranging from 3.04 to 4.27, which explained the phenotypic variation ranging from 5.59% to 12.87%. The alleles with negative effects are considered favorable alleles for the AC trait. Ten favorable alleles were detected over the entire population for the AC trait, with the PEV ranging from -0.45% to -4.32%.

The SSR loci simultaneously identified as responding to AC in more than two environments were RM6712 and RM6327. Among them, RM6712 (Chr.3: 35020004-35020027) was detected in four different environments (E1, E2, E3, and E5), with the maximum -LogP value of 4.27 in E1 and explained the corresponding maximum phenotypic variation of 8.83% in E1; it had the largest negative AAE^-^ of -4.87% in E3 (Table 4, Fig. 4c). Four RM6712 alleles were detected over the entire population (MAF>5%); RM6712-115bp and -95bp were associated with reducing the AC trait, while RM6712-85bp and -800bp were associated with increasing the AC trait. The favorable allele RM6712-115bp had the largest negative PEV (-4.32%) for reducing AC in E1 and its typical carrier accession is ‘Molingjing’ (Table 5).

RM6327 (Chr.11: 364257-364310) was detected in two environments (E2 and E3), displayed a maximum phenotypic variation of 12.87% in E2 and 11.39% in E3, and had negative AAE^-^ values of -2.66% and -2.75% in E2 and E3, respectively (Table 4, Fig. 4c). Nine alleles from RM6327 were detected over the entire population (MAF>5%). The favorable alleles including RM6327-230bp, -200bp, -190bp, -180bp, and -175bp were associated with reducing the AC trait, of which the RM6327-230bp allele in E2 showed the maximum negative phenotypic effect value (-3.78%) for reducing the AC; its typical cultivar is ‘Jinggunuo’ (Table 5).

For the AC trait, the SSR loci RM258 and RM5753 were identified in only one environment. RM258 (Chr.10: 17570591-17570612) was only detected in E1, with a maximum AAE^-^ of -5.28%, PVE of 5.69% and -LogP of 3.04 (Table 4, Fig. 4c). Three alleles from RM258 were detected over the entire population (MAF>5%). The favorable alleles including RM258-140bp, -135bp, and -125bp were associated with reducing the AC trait, of which the RM258-125bp allele showed the maximum negative PEV (-1.74%) for reducing the AC; its typical cultivar is ‘Yue109’ (Table 5).

RM5753 (Chr.6: 30452023-30452067), which was only detected in E2, had a PVE of 10.48% and - LogP value of 3.08 (Table 4, Fig. 4c). Six alleles were detected over the 462 accessions (MAF>5%). Four alleles, including RM5753-205bp, -200bp, -195bp, and 115bp, are associated with reducing the AC trait. Two alleles, including RM5753-135bp and -130bp, were associated with increasing the AC, of which the RM5753-195bp allele in E2 showed the maximum negative phenotypic effect value (-3.21%) for reducing the AC. Fifty accessions carried RM5753-195bp, with the typical cultivar being ‘Guozinuo’ (Table 5).

### The geographical distribution of variant alleles from the RM3600 and RM6327 loci detected among the 462 rice accessions

**Fig. 5:**
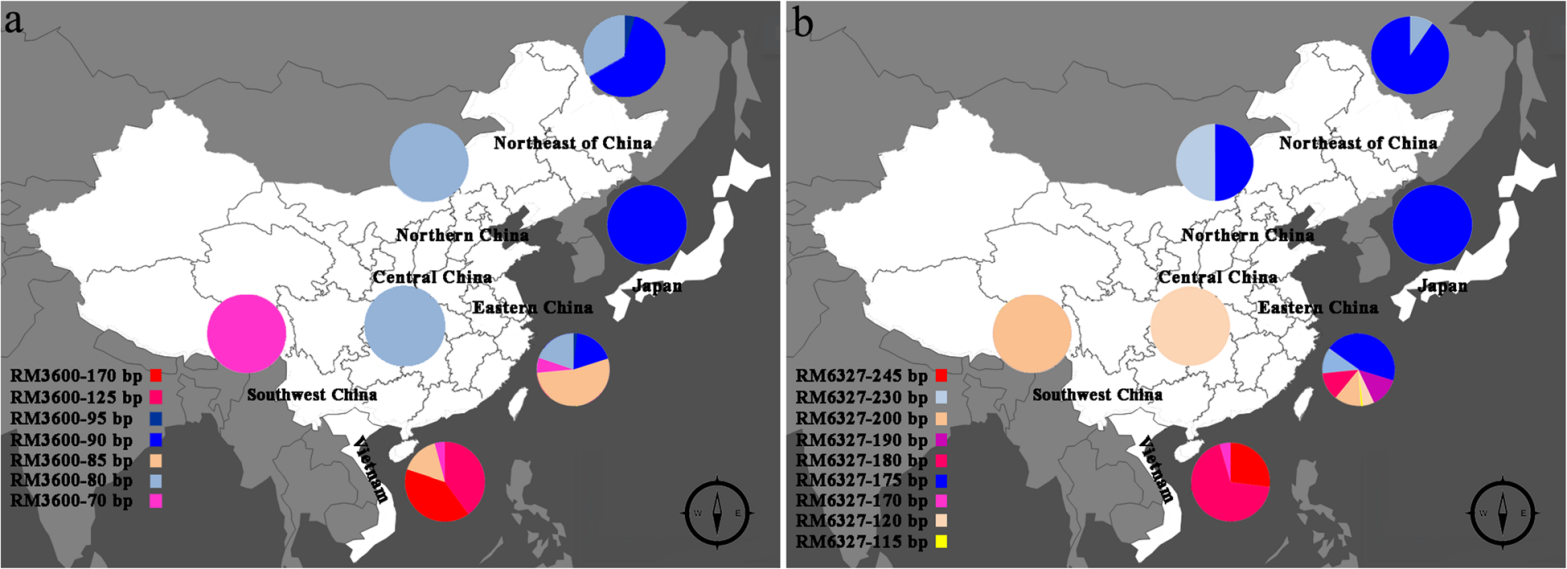
The geographic distribution of accessions carrying favorable alleles (The source map was taken from http://pixelmap.amcharts.com/). **a** Geographic distribution and the relative frequencies of SSR marker RM3600 alleles. **b** Geographic distribution and the relative frequencies of SSR marker RM6327 alleles. The color in the pie charts indicates the marker alleles within each locus status and geographic provenance of the germplasm category

We further analyzed the geographic distribution and the relative frequencies of alleles on the SSR loci associated with rice ECQs among the 462 accessions (Fig. 5a). RM3600 and RM6327 affected the rice ECQs and explained the maximum phenotypic variance of 10.12% and 12.87%, respectively. Therefore, these two SSR loci were selected for analysis.

According to their origins, the 462 cultivars were divided into the following 7 geographical regions; Eastern China, Northern China, Central China, Southwest of China, Northeast of China, Vietnam and Japan. RM3600-80bp, RM3600-85bp, RM3600-90bp and RM3600-95bp, which phenotypically reduced the GT, were frequently encountered with various origins including Central China, Eastern China, and Northern China; Vietnam and Eastern China; Japan, Eastern China, and Northeast of China; and Northeast of China, respectively. RM3600-70bp, RM3600-125bp and RM3600-170bp, which phenotypically increased the GT, originated in Southwest China, Vietnam, and Eastern China; Vietnam; and Vietnam, respectively. RM3600-90bp, which had the maximum PEV (2.00), was concentrated in carried accessions originating in Japan (100.0%) followed by Northeast of China (60.9%) and Eastern China (17.82%).

The rice accessions originating in Vietnam mainly carries the RM3600-70bp, -125bp, -170bp, and -85bp alleles. As rice was spread from south to Central China, the RM3600-125bp and RM3600-170bp alleles disappeared, while the RM3600-80bp, -90bp, and -95bp alleles appeared. Further north, the main rice accession alleles in Northern China and Japan were RM3600-90bp and -95bp, which are associated with reducing the GT trait.

The geographic distribution and relative frequencies of the 462 accessions carrying the RM6327 alleles are shown in Fig. 5b. RM6327-230bp, -200bp, -190bp, -180bp, and -175bp phenotypically reduced the AC and were frequently encountered Northeast of China and Northern China; Southwest China; Eastern China; Vietnam and Eastern China; and Northeast of China, Japan, Northern China and Eastern China, respectively. RM6327-245bp, -170bp, -120bp, and -115bp phenotypically increased the AC and originated in Vietnam, Vietnam, Central China, and Eastern China, respectively. The RM6327-175bp allele was concentrated in the carried accessions originating in northeast of China (60.9%) followed by Eastern China (38.2%), Northern China (20.0%) and Japan (11.1%).

The rice accessions that originated in Vietnam mainly carried the RM6327-245bp, -170bp, and -180bp alleles. As rice spread from south to Central China, the RM6327-245bp and -170bp alleles disappeared, while the RM6327-200bp, -230bp, -175bp, -120bp, -115bp, and -190bp alleles appeared. Further north, the main rice accession alleles in Northern China and Japan were RM6327-230bp and -175bp, which are associated with reducing the AC trait. Some alleles in this population disappeared with the northward movement of rice cultivation, though at the same time, additional alleles emerged to adapt to local ecological and geographical conditions and to meet the taste demands of people.

### Design for excellent parental combination

Fifteen excellent parental combinations were proposed for pyramiding favorable alleles into one single plant to improve rice ECQs (Table 6). Taking the GT trait as an example, when ‘Yebaodao’ was crossed with ‘Ligengqing’, all of the favorable alleles at the 4 loci could be combined into a single plant, ‘Yebaidao’ had 2 favorable alleles (RM267-95bp and RM3600-90bp) and ‘Ligengqing’ had 2 favorable alleles (RM232-145bp and RM264-195bp). Thus, the ASS of this variety could be theoretically increased to a grade of 4.8 (Table 6). All favorable alleles carried by the parents for rice ECQs are listed in Additional file 9: Table S7.

**Table 6:** Excellent parental combinations predicted for GT, GC and AC trait improvement

Traits	Parental combinations	No. of favorable alleles predicted	Total improvement predicted
GT (grade)	Yebaidao(2) × Ligengqing(2)	4	4.8
	Yebaidao(2) × Shiluqing(2)	4	4.8
	Kunnong8(1) × Yebaidao(3)	4	4.6
	Yebaidao(2) × Sanbailitou(2)	4	4.5
	Yebaidao(3) × Suzhouqing(1)	4	4.5
GC (mm)	Hongnong5(1) × Dongnongjingnuo418(1)	2	41.69
	Dongnongjingnuo418(1) × Baikenuo(1)	2	37.14
	Hongnong5(1) × Baikenuo(1)	2	36.69
	Baikenuo(1) × Shenlenuo(1)	2	35.14
	Baikenuo(1) × Haonuopie(1)	2	14.18
AC (%)	Baoxintaihuqing(2) × Yue109(2)	4	-9.07
	Baoxintaihuqing(3) × Jinggunuo(1)	4	-8.49
	Baoxintaihuqing(4) × Dongnongjingnuo418(2)	4	-8.49
	Baoxintaihuqing(3) × Hangzhounuo(2)	4	-8.49
	Baoxintaihuqing(2) × Baimangnuo(2)	4	-8.09

Digit in parentheses of the second column is the locus number

Some cultivars were found repeatedly in the supposed parental combinations; for example, ‘Yebaidao’ emerged 5 times in the combinations for the GT trait; ‘Baikenuo’, ‘Hongnong5’, and ‘Dongnongjingnuo418’ emerged of 4, 2, and 2 times, respectively for the GC trait; and ‘Baoxintaihuqing’ appeared in all combinations for the AC trait (Table 6), indicating that these cultivars possesses important values for breeding superior ECQs parents.

## Discussion

Without considering the population structure, there would have a high number of false positives in this association analysis. In this study, 462 rice accessions were classified into five subpopulations using a Structure model-based method (Fig. 2b), a dendrogram was created based on the Nei’s genetic distances (Fig. 2c), and the results from the PCA was consistent with the population structure based on these two collections (Fig. 2d). Therefore, the results from these three analyses are consistent. The results showed that accessions collected from Vietnam (latitudes < 17°N) were classified into POP1, while most accessions from northeastern China (latitudes ≥ 45°N) were classified into POP2. Thus, the geographic origin of accessions had an effect on the population structure. Different ecological environments and their corresponding unique geographical origins may be partly responsible for the genetic differentiation of the population, enabling germplasm accessions to adapt to the environment and the appearance of rare alleles.

Ten SSR loci were associated with rice ECQs from the entire set of accessions in this study, including 4 associated with GT, 2 associated with GC, and 4 associated with AC. Four of the 10 associations were in regions where the QTL associated with the ECQs had been identified and reported in previous studies using whole-genome marker resources from the Gramene website (http://www.gramene.org/), NCBI website (https://www.ncbi.nlm.nih.gov) and QTL database website (http://qtaro.abr.affrc.go.jp/), including RM267 and RM264 for GT and RM5753 for GC and AC [13, 19, 62, 63] (Table 7). Six loci were found for the first time in this study, including 2 for GT, 1 for GC and 3 for AC. For the 2 novel SSR loci for GT, RM3600 (Chr.9: 17107752-17107843) had the maximum PVE (10.12% in E2). The SSR locus RM6712 (Chr.3: 35020004-35020027) was a novel locus associated with GC, and it showed the largest -LogP value (3.3). For the 3 novel loci for AC, the average PVE ranged from 5.69% to 12.13% over the 5 environments (Table 4).

**Table 7:** Compared the SSR loci associated with GT, GC and AC in this study and QTLs for GT, GC and AC reported in the previous studies

Traits	NO.	SSR marker	Chr.	Start position (bp)^a^	End position (bp)^a^	QTL reported in the previous studies		
						Start position (bp)^b^	End position (bp)^b^	References
GT	1	RM232	3	8,409,404	8,410,886			
	2	RM267	5	21,881,317	21,881,455	26,848,154	28,906,633	Temnykh et al. (2001) [62]
	3	RM264	8	27,926,632	27,926,652	6,779,215	20,733,138	Septiningsih et al. (2003) [13]
	4	RM3600	9	17,107,752	17,107,843			
GC	1	RM6712	3	35,020,004	35,020,027			
	2	RM5753	6	30,452,023	30,452,067	2,686,204	4,897,779	Lanceras et al. (2000) [19]
AC	1	RM6712	3	35,020,004	35,020,027			
	2	RM5753	6	30,452,023	30,452,067	30,822,884	3,459,750	McCouch et al. (2002) [63]
	3	RM258	10	17,570,591	17,570,612			
	4	RM6327	11	364,257	364,310			

^a,b^The SSR Marker and the QTL physical position (bp) was inferred from the Gremene (http://www.gramene.org/markers) and NCBI (https://www.ncbi.nlm.nih.gov/Blast.cgi) respectively

The novel locus RM3600 (Chr.9: 17107752-17107843) for GT was detected close to the *Isoamylase3* (*ISA3*) gene (Chr.9: 17857347-17868663) that facilitates and affects starch metabolism in rice. To identify candidate genes to use as strong associated markers with LD analysis among the 262 SSR markers in this study, we found that RM210 (Chr.9: 19879785-19879804) was the closest marker to RM3600 in physical location; the distance between RM3600 and RM210 is approximately 2.7 Mb, while the *ISA3* gene is flanked by RM3600 and RM210. LD analysis was estimated by the *D′* values (*P* < 0.05), the intrachromosomal combinations of RM3600 and RM210 with a *D′* value of 0.76, and the minimum distance of LD decay (44.81 cM). The results indicated that RM3600 (-LogP=4.92) for GT was associated with *ISA3*. Additionally, association analyses for rice populations can provide an effective method for gene identification. The GWAS results in this study may increase our search for QTLs associated with the rice ECQs and provide useful information for further cloning.

The novel loci RM6712 (Chr.3: 35020004-35020027) and RM6327 (Chr.11: 364257-364310) simultaneously detected in more than 2 environments were considered as specific loci for AC. RM6712 was detected in four different environments (E1, E2, E3, and E5) with maximum -LogP values (4.27 in E1, 3.66 in E2, 3.22 in E3, and 3.17 in E5), which explained the corresponding maximum phenotypic variation of 8.83% in E1 and the largest negative AAE^-^ -4.87% in E3 (Table 4, Fig. 4c). RM6327 was detected in E2 and E3 with maximum PVE values (12.87% in E2, 11.39% in E3), -LogP values of 3.09 and 3.19, and negative AAE^-^ values of -2.66% and -2.75% in E2 and E3, respectively (Table 4, Fig. 4c).

In addition to the novel SSR loci detected in this study, four other SSR loci associated with rice ECQs were consistent with previous studies. RM264 on chromosome 8 had a PVE of 7.43% in E1 and RM267 on chromosome 5 associated with GT had a PVE of 7.04% in E3. These findings confirmed the results from Temnykh et al., who mapped microsatellite sequences in rice and reported that SSR loci RM264 and RM267 were associated with GT [62].

Similarly, Lanceras et al. (2000) reported that RM5753 on chromosome 6 was associated with the GC trait [19]. In this study, RM5753 (Chr.6: 30966850-30967050) was identified as co-associated with AC and GC; its physical distance is close to the *Wx* gene (Chr.6: 1765580-1770644) that encodes granule-bound starch synthase and controls GC and AC. RM506 (Chr.6: 435648-435677) was used to identify whether the *Wx* gene was strongly associated with RM5753, which is the closest marker to RM5753 based on physical location among the 262 SSR markers used in this study. The physical distance between the RM5753 and RM508 is approximately 30 Mb. The *Wx* gene is flanked by these two SSR markers and the intrachromosomal combinations of RM5753 and RM508 had a *D′* value of 1; the physical distance of this block was too long to determine whether the candidate gene was associated with RM5753. More markers on this chromosome should be further studied.

The favorable alleles were further mined using the results for the significant SSR loci associated with the rice ECQs obtained from the 462 accessions. Twenty-seven favorable alleles for rice ECQs were mined at 10 SSR loci. Among them, the favorable alleles were mainly carried by accessions collected from Eastern China, Vietnam and Northeast of China. For the AC trait, 55.79% of the favorable alleles were carried by the accessions collected from Eastern China, 31.14% were carried by accessions from Vietnam, and 9.67% were carried by accessions from Northeast of China. Similarly, the favorable alleles were detected in various accessions for GT and GC were consistent with these results. Based on the above results, during rice evolution and its progression from south to north, some alleles disappeared naturally or through artificial selection while others were retained in cultivars; moreover some appeared in modern cultivars for the first time.

For the GT trait, the broad-sense heritability average across the five environments for all 462 accessions was 95%, which was considerably high. Thus, marker-assisted selection (MAS) could obtain the expected results for improving GT. Among the four SSR loci identified for GT, RM3600 (Chr.9: 17107752-17107843) had the largest -LogP, PVE and AAE^+^ values at 4.92, 10.12% and 1.38, respectively. Among the three favorable alleles detected at this locus, RM3600-90 bp showed the largest positive phenotypic effect value (2.0 grade) for reducing GT. This favorable allele was carried by 86 accessions, and ‘Longdun105’ was a typical carrier material. We could improve GT greatly using the combinations described in Table 6.

For the GC trait, the broad-sense heritability average across the five environments for all 462 accessions was 94%. Among the two SSR loci associated with GC, RM5753 (Chr.6: 30452023-30452067) had the maximum PVE and positive AAE values of 10.54% and 20.52, respectively. Among the four favorable alleles found at this locus, RM5753-115bp showed the maximum positive phenotypic effect value (25.73 mm) for increasing the GC. Thirty-three accessions carried the RM5753-115bp allele and its typical cultivar is ‘Hongnong5’. GC could be improved by the combinations described in Table 6.

Among the three parameters measured for rice ECQs across the five environments, AC had the highest broad-sense heritability average of 98%. There were four SSR loci associated with AC, of which RM6327 (Chr.11: 364257-364310) had the maximum PVE (12.87% in E2, 11.39% in E3). Among the five negative alleles found at this locus, the favorable allele RM6327-230bp in E2 showed the maximum negative phenotypic effect value (-3.78%) for reducing the AC. Its typical cultivar is ‘Jinggunuo’. Thus, AC might be improved by the combinations described in Table 6.

Correlations and allele overlapping among the GT, GC and AC traits were observed; for example, GC was significantly negatively correlated with AC (-0.80**) and GT (-0.17**). Additionally, several cases showed that the same allele was significant for multiple traits. We observed RM5753 as coassociated with GC and AC, in which RM5753-195bp and RM5753-115bp simultaneously increased the phenotypic effect values for GC but reduced the PEV for AC (Table 5). RM6712 was also identified as coassociated with GC and AC, in which the favorable alleles RM6712-95bp and -115bp that increased the phenotypic effect values for GC overlapped with alleles that simultaneously reduced the PEV for AC (Table 5). The above results confirmed the results from He et al. (1999) and Yoko et al. (2015), who reported that GC and AC were both controlled by the *Wx* gene and displayed minor effects on the QTLs [10, 21]. Moreover, these overlapping alleles have the correct sign with respect to trait correlations, and illuminate the genetic basis of trait correlations.

We found that the geographical and ecological distribution of the experimental populations were sufficient with abundant genetic diversity. In total, 2462 alleles were detected with an average of 9.4 alleles per locus (Additional file 6: Table S5). Meanwhile, the proportion of rare alleles (allele frequency <5%) was 34.44% within the 2462 identified alleles. The slightly high ratio of rare alleles might have been caused by the wide distribution of latitudes for the natural population accessions; this result was consistent with the coefficient of variation for rice ECQs from 30.79% to 56.23%. As the rice cultivation area expanded from the plains to plateaus and from south to north, some new alleles appeared and some original alleles were lost, resulting in the emergence of accessions with rare alleles. Additionally, these results show that the natural population contains sufficient germplasm resources and genetic diversity, which are suitable for mining more favorable alleles for rice ECQs.

Analysis of the geographical distribution of the 7 alleles from RM3600 in the experimental natural population found that 76.44% of the favorable alleles (RM3600-90bp, -85bp, and -80bp) were carried by accessions collected from Eastern China (23°30’-38°23’N) and 10.37% of the favorable alleles were from Northeast of China (53°51’-53°33’N) accessions, but rarely found in the Vietnam (8°30’-23°22’N) accessions. In contrast, the RM3600-125bp and -170bp alleles were only found in the Vietnam accessions (Fig. 5a). We found that the accessions collected from Eastern China, Northeast of China and Japan were mainly japonica rice, which had a lower GT with the positive alleles. In contrast, the accessions collected from Vietnam and parts of the Lower Yangtze Region were mainly indica rice, which have a higher GT with negative alleles. Accessions collected from Eastern China and Vietnam carried abundant alleles and were to the Pearl River in Southern China, which implies that the geographic distribution of these alleles reflect the diffusion and diversification of Asian cultivated rice. Additionally, japonica rice was first domesticated in Southern China [64]. This finding also confirms the results from Khush (1997), who reported that Japonica rice moved north from South China and that artificial selection and adaptation from diverse ecological regions has resulted in cultivar diversity [65].

Similarly, we also studied the geographical distribution of the 9 RM6327 alleles in the experimental natural population. We found that 55.79% of the favorable alleles (RM6327-230bp, -175bp) were carried by accessions collected from Eastern China (23°30’-38°23’N) and 31.14% of the favorable alleles were from Northeast of China (53°51’-53°33’N) accessions, but only rarely (8.67%) found in Vietnam (8°30’-23°22’N) accessions. In contrast, the RM6327-245bp and -180bp alleles were mainly found in the Vietnam accessions, and rarely found in Eastern China (Fig. 5b). The accessions collected from the Vietnam (8°30’-23°22’N) had more types of alleles than those from Northeast of China (53°51’-53°33’N); the Vietnam materials were dominated by carriers of the three high frequency alleles RM6327-180bp (19.8%), -245bp (50.4%) and -200bp (3.3%) (Fig. 5b). This result is largely consistent with the report that Asian cultivated rice was domesticated from the tropical and subtropical mountains in Asia to high altitude and high latitude regions [64, 65].

Vietnam, Southwest of China and part of the Lower Yangtze Region mainly produce indica rice with a hard gel consistency, whereas Northeast China, Northern China, Central China and Japan mainly produced japonica rice with a soft gel consistency. This finding confirmed those reported by Wang et al. (2108), who reported that genetic differentiation of rice developed parallel to the development of ecological diversification and that long-term evolution, artificial selection and different ecological conditions contributed to the differentiation of Asian cultivated rice [66]. The above results revealed the proposed history of the spread of Asian cultivated rice in many parts of Southeast Asia. The expansion of rice cultivation eastwards from Southeast Asia was associated with artificial selection and different ecological conditions, which may have led to the favorable alleles that increased the gel consistency softness and adapted to local ecological conditions.

## Conclusions

There existed multiple gene loci underlying grain eating and cooking qualities in the natural population consisted of 462 accessions in rice. Among the 10 loci detected for grain GT, GC and AC, 6 loci were newly detected. Within the novel loci, the favorable allele RM3600-90bp on chromosome 9 could significantly reduce GT, RM6712-95bp on chromosome 3 could significantly increase GC, and RM6327-230bp on chromosome 11 could significantly reduce AC in hybrid japonica rice mixed rice samples.

## Additional files

Additional file 1: **Table S1.** Rice accessions geographical origin, their membership probabilities corresponding to each subpopulation, and the mean value of phenotypic data in five environments for GT, GC and AC. (XLSX 207 kb)

Additional file 2: **Table S2.** The test environments in which the 462 rice accessions population was evaluated. (DOCX 12 kb)

Additional file 3: **Figure S1.** Images showing measurements of amylose content with their materials using the automatic microplate spectrophotometer. (A) The automatic microplate spectrophotometer (TECAN Infinite 200 Pro, Austria). (B) ELISA plate with 96 holes. (C) The optical density value (OD) was showed in the computer monitor. (TIF 2316 kb)

Additional file 4: **Table S3.** Analysis of variance for GT, GC and AC traits of 462 rice accessions across 2011, 2012 and 2013 in Nanjing. *, **Significant at *P* ≤ 0.05 and 0.01, respectively. (DOC 31 kb)

Additional file 5: **Table S4.** Analysis of variance for GT, GC and AC of 462 rice accessions across Nanjing, Yuanyang and Xinyang in 2013. *, **Significant at *P* ≤ 0.05 and 0.01, respectively. (DOC 31 kb)

Additional file 6: **Table S5.** Summary statistics for the 262 SSR markers used in this study. (XLS 63 kb)

Additional file 7: **Table S6.** Analysis of molecular variance (AMOVA) for the five subpopulations of rice accessions. (DOCX 13 kb)

Additional file 8: **Figure S2.** Relationship between the D′ value and genetic distance of syntenic marker pairs in five subpopulations. (A) The subpopulation SP1. (B) The subpopulation SP2. (C) The subpopulation SP3. (D) The subpopulation SP4. (E) The subpopulation SP5. (TIF 3289 kb)

Additional file 9: **Table S7.** Favorable alleles carried by the superior parents for rice cooking quality traits and corresponding phenotypic effect value. (DOCX 18 kb)

## Abbreviations

${H}_B^2$: Broad-sense heritability
AAE: Average allele effect
AC: Amylose content
ANOVA: Analysis of variance
ASS: Alkali spreading score
FDR: False discovery rate
F_*ST*_: Coefficient of genetic differentiation
GC: Gel consistency
GLM: General linear model
GT: Gelatinization temperature
LD: Linkage disequilibrium
MAF: Minimum allele frequency
MLM: Mixed linear model
PCA: Principal components analysis
PIC: Polymorphism information content
PVE: Phenotypic variation explained
SSR: Simple sequence repeat

## References

[1] Muthayya S, Sugimoto JD, Montgomery S, Maberly G. An overview of global rice production, supply, trade, and consumption. Ann N Y Acad Sci. 2014; 1324:7-14. https ://10.1111/nyas.12540 25224455

[2] Yuan L (2014). Development of Hybrid Rice to Ensure Food Security. Rice Science..

[3] Hong D, Leng Y (2004). Genetic analysis of heterosis for number of spikelets per panicle and panicle length of F_1_ hybrids in japonica rice hybrids. Chin J Rice Sci.

[4] Hua ZT, Wang YR. Advances in japonica hybrid rice breeding. In: Xie F, Hardy B, editors. Accelerating hybrid rice development. Los Baños (Philippines): International Rice Research Institute. 2009. p. 139-49.

[5] Khush GS, Kumar I, Virmani SS. Grain quality of hybrid rice. In: IRRI, editor. Hybrid Rice. Manila (Philippines): International Rice Research Institute. 1988. p. 201–15.

[6] Tan Y, Li J, Yu S, Xing Y, Xu C, Zhang Q (1999). The three important traits for cooking and eating quality of rice grains are controlled by a single locus in an elite rice hybrid, Shanyou 63. Theor Appl Genet..

[7] Shi C, Zhu J, Zang R, Chen G (1997). Genetic and heterosis analysis for cooking quality traits of indica rice in different environments. Theor Appl Genet..

[8] Delwiche S, Mckenzie K, Webb B (1996). Quality characteristics in rice by near-infrared reflectance analysis of whole-grain milled samples. Cereal Chem..

[9] Zhang G, Cheng Z, Zhang X, Guo X, Su N, Jiang L (2011). Double repression of soluble starch synthase genes SSIIa and SSIIIa in rice (*Oryza sativa* L.) uncovers interactive effects on the physicochemical properties of starch. Genome..

[10] He P, Li S, Qian Q, Ma Y, Li J, Wang W (1999). Genetic analysis of rice grain quality. Theor Appl Genet..

[11] Gao Z, Zeng D, Cui X, Zhou Y, Yan M, Huang D (2003). Map-based cloning of the ALK gene, which controls the gelatinization temperature of rice. Science in China..

[12] Fan C, Yu X, Xing Y, Xu C, Luo L, Zhang Q (2005). The main effects, epistatic effects and environmental interactions of QTLs on the cooking and eating quality of rice in a doubled-haploid line population. Theor Appl Genet..

[13] Septiningsih E, Trijatmiko K, Moeljopawiro S, Mccouch S (2003). Identification of quantitative trait loci for grain quality in an advanced backcross population derived from the *Oryza sativa* variety IR64 and the wild relative *O. rufipogon*. Theor Appl Genet..

[14] Aluko G, Martinez C, Tohme J, Castano C, Bergman C, Oard JH (2004). QTL mapping of grain quality traits from the interspecific cross *Oryza sativa* × *O. glaberrima*. Theor Appl Genet..

[15] Xu F, Bao J, He Q, Park Y (2016). Genome-wide association study of eating and cooking qualities in different subpopulations of rice (*Oryza sativa* L.). BMC Genomics..

[16] Nakamura Y, Francisco P, Hosaka Y, Sato A, Sawada T, Kubo A (2005). Essential amino acids of starch synthase IIa differentiate amylopectin structure and starch quality between japonica and indica rice varieties. Plant Mol Biol..

[17] Tang S, Khush G, Juliano B (1991). Genetics of gel consistency in rice (*Oryza sativa* L.). J Genet..

[18] Li J, Xiao J, Silvana G, Jiang L, Wan Y, Deng Q (2004). QTL detection for rice grain quality traits using an interspecific backcross population derived from cultivated Asian (*O. sativa* L.) and African (*O. glaberrima* S.) rice. Genome.

[19] Lanceras J, Huang Z, Naivikul O, Vanavichit A, Ruanjaichon V, Tragoonrung S (2000). Mapping of genes for cooking and eating qualities in Thai jasmine rice (KDML105). DNA Res..

[20] Kiswara G, Lee JH, Hur YH, Cho JH, Lee JY, Kim SY (2014). Genetic analysis and molecular mapping of low amylose gene *du12*(t) in rice (*Oryza Sativa* L.). Theor Appl Genet..

[21] Yoko T, Hiroki M, Keitaro S, Hideyuki H, Osamu I, Noriaki A (2015). *qAC2*, a novel QTL that interacts with *Wx* and controls the low amylose content in rice (*Oryza sativa* L.). Theor Appl Genet.

[22] Sano Y (1984). Differential regulation of waxy gene expression in rice endosperm. Theor Appl Genet..

[23] Isshiki M, Nakajima M, Satoh H, Shimamoto K (2000). dull: rice mutants with tissue-specific effects on the splicing of the waxy pre-mRNA. Plant..

[24] Isshiki M, Matsuda Y, Tasaki A, Wong HL, Satoh H, Shimamoto K (2008). Du3, a mRNA cap-binding protein gene, regulates amylose content in japonica rice seeds. Plant Biotechnol..

[25] Satoh H, Omura T (1981). New endosperm mutations induced by chemical mutagen in rice, *Oryza sativa* L. Jpn J Breed..

[26] Yano M, Okuno K, Satoh H, Omura T (1988). Chromosomal location of genes conditioning low amylose content of endosperm starches in rice, *Oryza sativa* L. Theor Appl Genet..

[27] Satoh H, Omura T (1986). Mutagenesis in rice by treating fertilized egg cells with nitroso compounds. Rice Genetics. Proceeding of the International Rice Genetics Symposium.

[28] Pooni H, Kumar I, Khush G (1992). A comprehensive model for disomically inherited metrical traits expressed in triploid tissues. Heredity..

[29] Wang W, Mauleon R, Hu Z, Chebotarov D, Tai S, Wu Z (2018). Genomic variation in 3,010 diverse accessions of Asian cultivated rice. Nature..

[30] Fan Z, Spinka C, Jin L, Jung S (2005). Pedigree linkage disequilibrium mapping of quantitative trait loci. Eur J Hum Genet..

[31] Mo H (1989). Genetic Models and Generation Means for Endosperm Traits. J Genet..

[32] Mo H (1995). Identification of genetic control for endosperm traits in cereals. J Genet..

[33] Xu C, Mo H, Zhong A, Zhu Q (1995). Genetical control of quality traits of rice grains in indica-japonica hybrids. J Genet..

[34] Bao J, Wu Y, Hu B, Wu P, Cui H, Shu Q (2002). QTL for rice grain quality based on a DH population derived from parents with similar apparent amylose content. Euphytica.

[35] Cardon L, Bell J (2001). Association study designs for complex diseases. Nat Rev Genet..

[36] Zondervan K, Cardon L (2004). The complex interplay among factors that influence allelic association. Nat Rev Genet..

[37] Flint S, Thornsberry M, Buckler V (2003). Structure of linkage disequilibrium in plants. Annu Rev Plant Biol..

[38] Gupta P, Rustgi S, Kulwal P (2005). Linkage disequilibrium and association studies in higher plants: Present status and future prospects. Plant Mol Biol..

[39] Yu J, Pressoir G, Briggs W, Bi I, Yamasaki M, Doebley J (2005). A unified mixed-model method for association mapping that accounts for multiple levels of relatedness. Nat Genet..

[40] Price A, Zaitlen N, Reich D, Patterson N (2010). New approaches to population stratification in genome-wide association studies. Nat Rev Genet..

[41] Clayton D, Walker N, Smyth D, Pask R, Cooper J, Maier L (2005). Population structure, differential bias and genomic control in a large-scale, case-control association study. Nat Genet.

[42] Segura V, Vilhjálmsson B, Platt A, Korte A, Seren Ü, Long Q (2012). An efficient multi-locus mixed-model approach for genome-wide association studies in structured populations. Nat Genet..

[43] Borba T, Brondani R, Breseghello F, Coelho A, Mendonca J, Rangel P (2010). Association mapping for yield and grain quality traits in rice (*Oryza sativa* L.). Genet Mol Biol..

[44] Dang X, Thi T, Dong G, Wang H, Edzesi W (2014). Genetic diversity and association mapping of seed vigor in rice (*Oryza sativa* L.). Planta.

[45] Sowadan O, Li D, Zhang Y, Zhu S, Hu X, Bhanbhro L, et al. Mining of favorable alleles for lodging resistance traits in rice (*Oryza sativa*) through association mapping. Planta. 2018. 10.1007/s00425-018-2885-y. 10.1007/s00425-018-2885-y 29637263

[46] Liu E, Zeng S, Chen X, Dang X, Liang L, Wang H (2017). Identification of putative markers linked to grain plumpness in rice (*Oryza sativa* L.) via association mapping. BMC Genet.

[47] Little R, Hilder G, Dawson E (1958). Differential effect of dilute alkali on 25 varieties of milled white rice. Cereal Chem..

[48] Jennings P, Coffman W, Kauffman H (1979). Rice improvement.

[49] Cagampang G, Perez C, Juliano B (1973). A gel consistency test for the eating quality of rice. Sci Food Agric..

[50] Zhu X, Shen S, Chen W, Shu Q (2004). Application of microplate spectrophotometer in determination of apparent amylose content and mutant selection in rice. Acta Agriculturae Nucleatae Sinica.

[51] Wang L, Liu W, Xu Y, He Y, Luo L, Xing Y (2007). Genetic basis of 17 traits and viscosity parameters characterizing the eating and cooking quality of rice grain. Theor Appl Genet..

[52] Tai T, Tanksley S (1990). A rapid and inexpensive method for isolation of total DNA from dehydrated plant tissue. Plant Mol Biol Report.

[53] Falush D, Stephens M, Pritchard J (2003). Inference of population structure using multilocus genotype data: linked loci and correlated allele frequencies. Genetics.

[54] Evanno G, Regnaut S, Goudet J (2005). Detecting the number of clusters of individuals using the software STRUCTURE: a simulation study. Mol Ecol..

[55] Liu K, Muse S (2005). PowerMarker: an integrated analysis environment for genetic marker analysis. Bioinformatics..

[56] He J, Meng S, Zhao T (2017). An innovative procedure of genome-wide association analysis fits studies on germplasm population and plant breeding. Theor Appl Genet.

[57] Weir B, Hill W (2002). Estimating F-statistics. Annu Rev Genet..

[58] Excoffier L, Lischer H (2015). Arlequin ver. 3.5: an integrated software package for population genetics data analysis. Evol Bioinform Online..

[59] Bradbury P, Zhang Z, Kroon D, Casstevens T, Ramdoss Y, Buckler E (2007). TASSEL: software for association mapping of complex traits in diverse samples. Bioinformatics.

[60] Muralidharan O (2010). An empirical Bayes mixture method for effect size and false discovery rate estimation. Ann Appl Stat..

[61] Breseghello F, Sorrells M (2006). Association mapping of kernel size and milling quality in wheat (*Triticum aestivum* L.) cultivars. Genetics..

[62] Temnykh S, DeClerck G, Lukashova A, Lipovich L, Cartinhour S, McCouch S (2001). Computational and experimental analysis of microsatellites in rice (*Oryza sativa* L.): Frequency, Length Variation, Transposon Associations, and Genetic Marker Potential. Genome Res.

[63] Mccouch S, Teytelman L, Xu Y, Lobos K, Clare K (2002). Development and mapping of 2240 new SSR markers for rice (*Oryza sativa* L.). DNA Res.

[64] Huang X, Kurata N, Wei X, Wang Z, Wang A, Zhao Q (2012). A map of rice genome variation reveals the origin of cultivated rice. Nature..

[65] Khush G (1997). Origin, dispersal, cultivation and variation of rice. Plant Mol Biol.

[66] Wang J, Wan X, Li H, Pfeiffer W, Crouch J, Wan J (2007). Application of identified QTL-marker associations in rice quality improvement through a design-breeding approach. Theor Appl Genet..

