# Supplementary material for: Favorable alleles mining for gelatinization temperature, gel consistency and amylose content in Oryza sativa by association mapping
Source: BMC Genet. 2019 Mar 19;20:34. doi: 10.1186/s12863-019-0735-y (PMC6423859; doi:10.1186/s12863-019-0735-y)
Supplement: Supplementary file 2 — Table S2. The test environments in which the 462 rice accessions population was evaluated. (DOCX 12 kb) [file 12863_2019_735_MOESM2_ESM.docx]

Supplementary table S2 The test environments in which the 462 rice accessions population was evaluated

|  | Year | | | Site | |  |
| --- | --- | --- | --- | --- | --- | --- |
| E1 | May–October,2011 | | | Nanjing Agricultural University experiment farm, Jiangsu, China. | | |
|  |  | | | 32°7''N, 118°4'E |  |  |
| E2 | May–October,2012 | | | Nanjing Agricultural University experiment farm, Jiangsu, China. | | |
|  |  |  |  | 32°7''N, 118°4'E |  |  |
| E3 | May–October,2013 | | | Nanjing Agricultural University experiment farm, Jiangsu, China. | | |
|  |  |  |  | 32°7''N, 118°4'E |  |  |
| E4 | May–November,2013 | | | Yuanyang Farm , Henan, China. | |  |
|  |  |  |  | 35°5"N, 113°90'6"E |  |  |
| E5 | May–November,2013 | | | Xinyang Farm, Henan, China. | |  |
|  |  |  |  | 32°10'N, 114°10'2''E |  |  |
