# Supplementary figures and images for: Favorable alleles mining for gelatinization temperature, gel consistency and amylose content in Oryza sativa by association mapping
Source: BMC Genet. 2019 Mar 19;20:34. doi: 10.1186/s12863-019-0735-y (PMC6423859; doi:10.1186/s12863-019-0735-y)

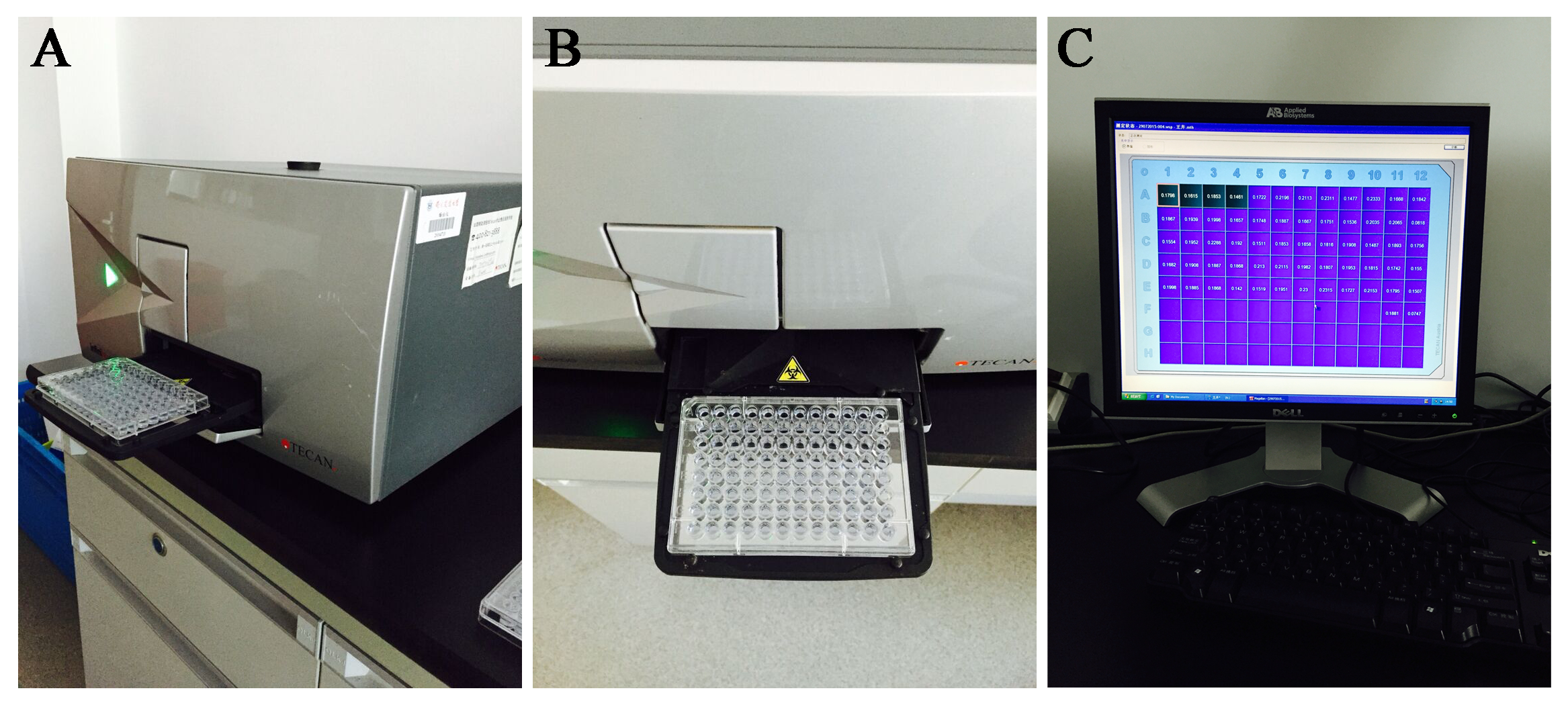

Supplement: Supplementary file 3 — Figure S1. Images showing measurements of amylose content with their materials using the automatic microplate spectrophotometer. (A) The automatic microplate spectrophotometer (TECAN Infinite 200 Pro, Austria). (B) ELISA plate with 96 holes. (C) The optical density value (OD) was showed in the computer monitor. (TIF 2316 kb) [file 12863_2019_735_MOESM3_ESM.tif]

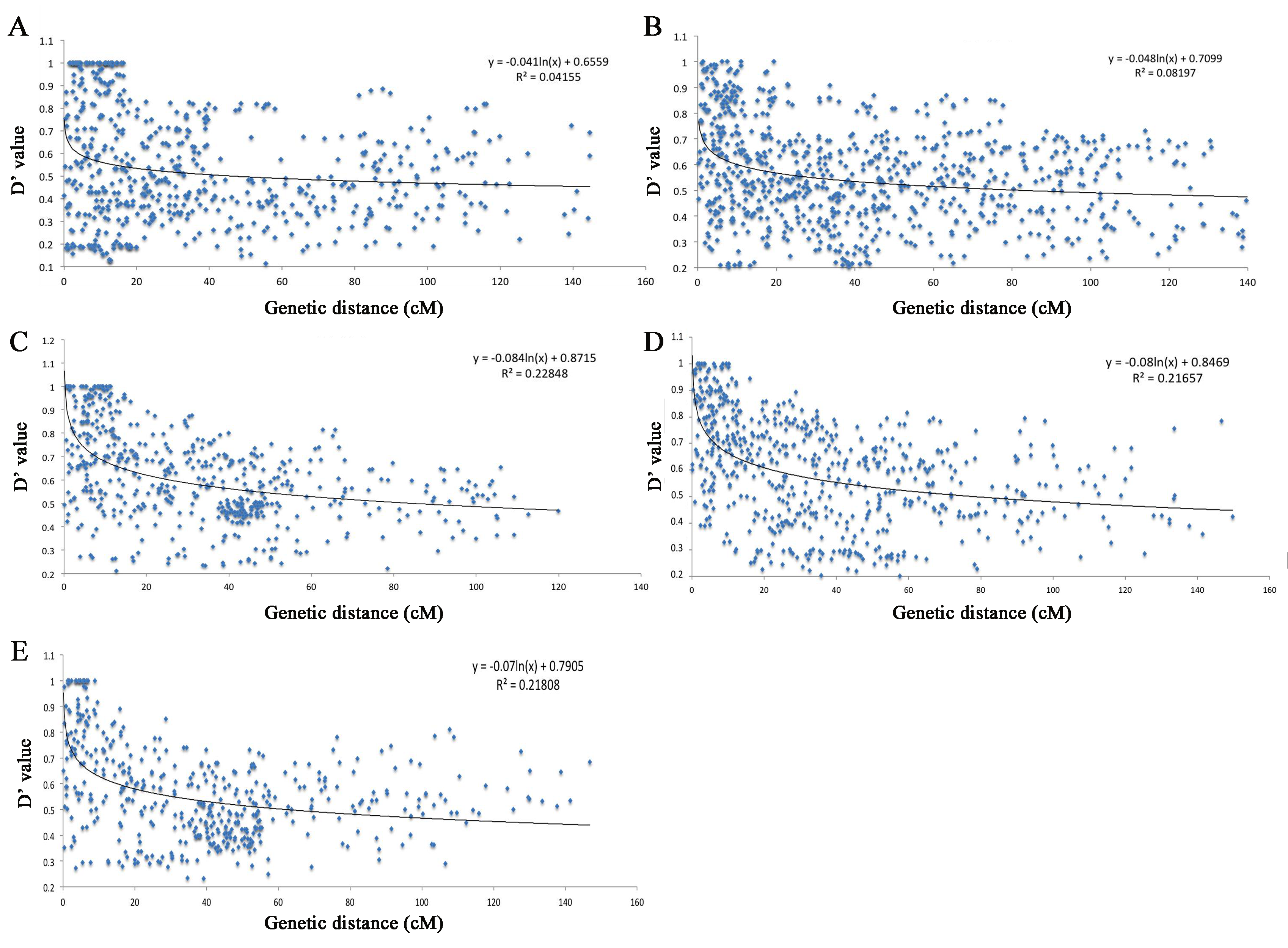

Supplement: Supplementary file 8 — Figure S2. Relationship between the D′ value and genetic distance of syntenic marker pairs in five subpopulations. (A) The subpopulation SP1. (B) The subpopulation SP2. (C) The subpopulation SP3. (D) The subpopulation SP4. (E) The subpopulation SP5. (TIF 3289 kb) [file 12863_2019_735_MOESM8_ESM.tif]
