# Supplementary material for: Favorable alleles mining for gelatinization temperature, gel consistency and amylose content in Oryza sativa by association mapping
Source: BMC Genet. 2019 Mar 19;20:34. doi: 10.1186/s12863-019-0735-y (PMC6423859; doi:10.1186/s12863-019-0735-y)
Supplement: Supplementary file 4 — Table S3. Analysis of variance for GT, GC and AC traits of 462 rice accessions across 2011, 2012 and 2013 in Nanjing. *, **Significant at P ≤ 0.05 and 0.01, respectively. (DOC 31 kb) [file 12863_2019_735_MOESM4_ESM.doc]

**Supplementary table S3** Analysis of variance for GT, GC and AC traits of 462 rice accessions across 2011, 2012 and 2013 in Nanjing.

| Source | *df* | GT | | GC | | AC | |
| --- | --- | --- | --- | --- | --- | --- | --- |
|  |  | MS | *F* values | MS | *F* values | MS | *F* values |
| Among years | 2 | 10.42 | 74.56** | 11293.34 | 50.33** | 717.29 | 46.85** |
| Among replications within years | 3 | 0.14 | 0.83 | 224.4 | 7.89** | 15.31 | 10.341** |
| Among genotypes | 461 | 12.36 | 73.84** | 3039.1 | 106.91** | 178.6 | 120.64** |
| Interactions between years and genotypes | 922 | 2.68 | 15.98** | 355.29 | 12.50** | 17.5 | 11.82** |
| Error | 1386 | 0.17 |  | 28.85 |  | 1.51 |  |

*, **Significant at P≤0.05 and 0.01, respectively
