# Supplementary material for: Favorable alleles mining for gelatinization temperature, gel consistency and amylose content in Oryza sativa by association mapping
Source: BMC Genet. 2019 Mar 19;20:34. doi: 10.1186/s12863-019-0735-y (PMC6423859; doi:10.1186/s12863-019-0735-y)
Supplement: Supplementary file 5 — Table S4. Analysis of variance for GT, GC and AC of 462 rice accessions across Nanjing, Yuanyang and Xinyang in 2013. *, **Significant at P ≤ 0.05 and 0.01, respectively. (DOC 31 kb) [file 12863_2019_735_MOESM5_ESM.doc]

**Supplementary table S4** Analysis of variance for GT, GC and AC traits of 462 rice accessions across Nanjing, Yuanyang and Xinyang in 2013.

| Sources of variation | *df* | GT | | GC | | AC | |
| --- | --- | --- | --- | --- | --- | --- | --- |
|  |  | MS | *F* values | MS | *F* values | MS | *F* values |
| Among sites | 2 | 245.8 | 1435.21** | 7004.55 | 81.91** | 672.06 | 11.02* |
| Among replications within sites | 3 | 0.17 | 1.12 | 85.52 | 2.01 | 60.96 | 543.90** |
| Among genotypes | 461 | 11.62 | 75.93** | 3012.41 | 70.86** | 190.74 | 1701.69** |
| Interactions between sites and genotypes | 922 | 3.12 | 20.37** | 311.18 | 7.32** | 15.95 | 142.28** |
| Error | 1386 | 0.15 |  | 42.6 |  | 0.24 |  |

*, **Significant at P≤0.05 and 0.01, respectively
