# Supplementary material for: Favorable alleles mining for gelatinization temperature, gel consistency and amylose content in Oryza sativa by association mapping
Source: BMC Genet. 2019 Mar 19;20:34. doi: 10.1186/s12863-019-0735-y (PMC6423859; doi:10.1186/s12863-019-0735-y)
Supplement: Supplementary file 7 — Table S6. Analysis of molecular variance (AMOVA) for the five subpopulations of rice accessions. (DOCX 13 kb) [file 12863_2019_735_MOESM7_ESM.docx]

**Supplementary Table S6** Analysis of molecular variance (AMOVA) for the five subpopulations of rice accessions

| Source of variation | Dgree of freedom | Sum of squares | Variance components | Percentage of variation | *P* value |
| --- | --- | --- | --- | --- | --- |
| Among subpopulations | 4 | 16419.26 | 18.75 | 34.48 | ＜0.001 |
| Among individuals within subpopulations | 457 | 33177.38 | 35.62 | 65.52 | ＜0.001 |
| Total | 461 | 49596.64 | 54.37 |  |  |
