# Supplementary material for: Favorable alleles mining for gelatinization temperature, gel consistency and amylose content in Oryza sativa by association mapping
Source: BMC Genet. 2019 Mar 19;20:34. doi: 10.1186/s12863-019-0735-y (PMC6423859; doi:10.1186/s12863-019-0735-y)
Supplement: Supplementary file 9 — Table S7. Favorable alleles carried by the superior parents for rice cooking quality traits and corresponding phenotypic effect value. (DOCX 18 kb) [file 12863_2019_735_MOESM9_ESM.docx]

Supplementary Table S7 Favorable alleles carried by the superior parents for rice cooking quality traits and corresponding phenotypic effect value

| Traits | Superior parents | Locus- allele ( corresponding phenotypic effect value) |
| --- | --- | --- |
| GT | Sanbailitou | RM232-145(0.67)，RM3600-90(2.00) |
|  | Souzhouqing | RM232-145(0.67)，RM3600-85(1.02) |
|  | Ligengqing | RM232-145(0.67)，RM3600-85(1.02)，RM264-195(0.97) |
|  | Laowusi | RM232-150(0.03)，RM3600-85(1.02) |
|  | Feilaifeng | RM232-155(0.63)，RM267-125(0.61)，RM3600-90(2.00)， RM264-130(0.74) |
|  | Xiangnuodao | RM267-95(1.11)， RM3600-90(2.00)，RM264-130(0.74) |
|  | Songjing12 | RM232-145(0.67)，RM3600-80(0.29) |
|  | Longdun105 | RM232-150(0.03)，RM3600-90(2.00) |
|  | wanyedao | RM232-145(0.67)，RM3600-85(1.02) |
|  | Kuobanzhong | RM267-120(0.39)，RM3600-80(0.29) |
|  | Shiluqing | RM232-145(0.67)，RM3600-85(1.02)，RM264-195(0.97) |
|  | Kunnong8 | RM232-150(0.03)，RM267-125(0.61)，RM3600-85(1.02)，RM264-160(0.85) |
|  | Yebaidao | RM232-155(0.63)，RM267-95(1.11)，RM3600-90(2.00)，RM264-130(0.74) |
| GC | Dongnongjingnuo418 | RM6712-95(13.12) |
|  | Baikenuo | RM6712-115(11.12)，RM5753-195(24.02) |
|  | Shenlenuo | RM5753-195(24.02) |
|  | Hongnong5 | RM5753-115(28.57) |
|  | Haonuopie | RM5753-130(3.06) |
| AC | Molingjing | RM6712-115(-2.20)，RM258-135(-0.70) |
|  | Xudao3 | RM6712-115(-2.20)，RM258-135(-0.70)，RM6327-175(-1.92) |
|  | Wujing15 | RM6712-115(-2.20)，RM258-135(-0.70)，RM6327-175(-1.92) |
|  | Baoxintaihuqing | RM6712-115(-2.20)，RM5753-195(-3.21)，RM258-140(-0.76)，RM6327-175(-1.92) |
|  | Dongnongjingnuo418 | RM6712-95(-1.68)，RM6327-230(-2.32) |
|  | Munian4 | RM6712-95(-1.68)，RM258-135(-0.70) |
|  | Hangzhounuo | RM6712-95(-1.68)，RM6327-230(-2.32) |
|  | Guozinuo | RM5753-195(-3.21)， RM258-140(-0.76)，RM6327-175(-1.92) |
|  | Suyunuo | RM5753-115(-2.21)，RM258-135(-0.70) |
|  | Suzhouqing | RM5753-205(-1.13)，RM258-140(-0.76)，RM6327-175(-1.92) |
|  | Yue109 | RM258-125(-1.74)，RM6327-175(-1.92) |
|  | Wandao98 | RM258-140(-0.76) |
|  | Shenlenuo | RM5753-195(-3.21)，RM258-135(-0.70) |
|  | Jinggunuo | RM6712-95(-1.68)，RM5753-205(-1.13)，RM6327-230(-2.32) |
|  | Baimangnuo | RM5753-205(-1.13)，RM258-140(-0.76)，RM6327-175(-1.92) |
